# Factors associated with failures and outcomes of Peripherally Inserted Central Catheters in hospitalized COVID-19 patients

**DOI:** 10.1590/1518-8345.8298.4970

**Published:** 2026-10-23

**Authors:** Amanda Saba, Jéssica Anastácia Silva Barbosa

**Affiliations:** 1 Universidade de São Paulo, São Paulo, SP, Brazil.; 2 Sociedade Beneficente de Senhoras Hospital Sírio-Libanês, São Paulo, SP, Brazil.

**Keywords:** Indwelling Catheters, Nursing Care, COVID-19, Catheter-Related Infections, Equipment Failure, Inpatients.

## Abstract

**Objective:**

to analyze factors associated with catheter progression failure and outcomes of Peripherally Inserted Central Catheters inserted by nurses in hospitalized COVID-19 patients.

**Method:**

observational cohort study was conducted with hospitalized COVID-19 patients, following the STROBE guidelines. Categorical variables were described as absolute and relative frequencies; quantitative variables were expressed as median and interquartile range. Non-parametric tests were applied due to non-normal distribution. Associations were analyzed using Pearson’s chi-squared or Fisher’s exact tests. Logistic regression was performed for variables associated with outcomes.

**Results:**

a total of 1061 Peripherally Inserted Central Catheters were analyzed. The main indications were for antibiotic therapy, and most catheters were removed after treatment completion. First-attempt failure and clinical patient profile were associated with catheter progression failure. Subcutaneous heparin was a protective factor. Among Peripherally Inserted Central Catheters successfully inserted, tip confirmation via chest X-ray and catheter-to-vessel ratio between 31%-40% were associated with thrombosis, whereas Sherlock™ confirmation was protective.

**Conclusion:**

catheter progression failure was associated with clinical conditions and first-attempt failure. Adverse outcomes were associated with X-ray tip confirmation and catheter-to-vessel ratio, whereas Sherlock™ use and subcutaneous heparin were protective factors.

## Highlights

**(1)**First-attempt failure was associated with progression failure.

**(2)**Subcutaneous heparin is associated with reduced PICC progression failure.

**(3)**The method used for tip confirmation can be a protective factor for thrombosis.

**(4)**11% of PICCs removed for SBSI had CLABSI, highlighting need to detect BSI.

## Introduction

Peripherally Inserted Central Catheter (PICC) presents an alternative to the Central Venous Catheter (CVC) and is widely used for medium- and long-term treatments in both inpatient and outpatient settings^1^. During the coronavirus disease 2019 (COVID-19) pandemic, patients-particularly those in the intensive care unit (ICU)-often required reliable vascular access for supportive therapy and blood sampling^2^. This situation underscored the need for efficient resource allocation and optimization of healthcare strategies during the pandemic^3^.

A critical challenge during this time was ensuring safe and effective vascular access while minimizing the risk of airborne contamination for healthcare workers. The GaVeCelt Consensus Statement (*Gli Accessi Venosi Centrali a Lungo Termine*) discusses the advantages of using PICCs over CVCs in patients with severe respiratory difficulties, such as those receiving continuous positive airway pressure (CPAP) or non-invasive ventilation (NIV), and those who are prone or anticoagulated. PICCs have been shown to reduce the risk of pleuropulmonary complications, avoid interference with respiratory management, and lower the risk of exit-site infections and operator exposure to tracheal secretions, particularly in tracheostomized patients^4^.

Another key benefit of PICCs is their insertion under ultrasound guidance which avoids the need for X-rays when possible and reduces the risk of airborne contamination. This is particularly beneficial for bedside insertion, eliminating the need to move patients and thereby reducing exposure to the virus for healthcare professionals^2^. Moreover, the use of Intracavitary Electrocardiogram (IC-ECG)-guided PICC placement offers real-time tip confirmation during insertion, making it more accurate, safer, and cost-effective than traditional X-ray guidance^5^ ^-^ ^6^. IC-ECG guidance also eliminates the delays associated with X-ray imaging, streamlining catheter use^3^.

Despite these advantages, PICCs are not without their limitations. For example, ECG-guided PICC placement may not be suitable for patients with certain ECG changes such as atrial fibrillation or those with pacemakers^5^. In these cases, an X-ray may still be required to confirm catheter placement before use. Additionally, factors like therapy type, duration, and vessel conditions must be carefully considered when selecting the most appropriate device for each patient^5^. PICC removal typically occurs once intravenous therapy concludes, but complications such as bloodstream infections, venous thrombosis, or occlusions may require premature removal. In a French study, 38% of catheters were removed before completing the intravenous therapy^7^. Preventative measures during insertion, including the use of aseptic technique, maximal barrier protection, and ultrasound guidance, can minimize these risks. Ongoing catheter maintenance also requires continuous education and training of healthcare providers, particularly regarding adherence and proper flushing techniques and appropriate dressing changes^8^.

Critically ill COVID-19 patients, who are prone to alterations in the coagulation cascade and hypercoagulation, present a high risk of thrombotic events. This increases the complexity of vascular access and makes CVCs less ideal due to the risk of peri-catheter thrombosis and additional complications like pneumothorax^9^ ^-^ ^10^.

While the acute phase of the pandemic has passed, long-term COVID-19 complications and the ongoing care of patients affected by the virus highlight the need for further research into vascular access strategies. 

Despite the well-established benefits of PICCs and the extensive literature addressing post-insertion complications, less attention has been given to events occurring during the insertion process itself. Specifically, when catheter progression failure-when the catheter does not advance fully, even after maneuvers-occurs before the device reaches its intended intravascular position. This failure not only delays treatment but can also increase costs and may require additional interventions. Given the ongoing challenges and the need for evidence-based decision-making in catheter management for COVID-19 patients, this study analyzed outcomes of Peripherally Inserted Central Catheters (PICCs) inserted by nurses in hospitalized COVID-19 patients and factors associated with catheter progression failure.

## Methods

### Design

A cohort observational study was conducted following the Strengthening the Reporting of Observational Studies in Epidemiology (STROBE) guidelines^11^. 

### Study settings

This study was conducted at a high-complexity teaching and research hospital located in the city of São Paulo, accredited by the Joint Commission International (JCI) and with other certifications. This hospital responds to emergencies with various levels of complexity, providing care for patients with private health insurance plans or in the context of public-private partnerships. The hospital has a vascular access specialized team (VAST) composed of registered nurses who insert and maintain PICCs, ultrasound-guided peripheral intravenous catheters, weekly PICC assessment, among other activities. VAST had seven members and hired other three professionals due to high demand during the pandemic. All members had qualification certificate for PICC insertion and specialization degree, while three members held masters’ degree.

### Participants

The study population consisted of hospitalized COVID-19 patients with PICC insertion, aged 18 years or older. Outpatients and oncology patients, and PICC placed by vascular doctors were excluded from this study. PICC records with incomplete outcome information were excluded from the final sample. For patients with more than one PICC placement, all PICC placements were considered.

### PICC request and insertion

The medical team assesses intravenous therapy (duration and components) and patient access conditions to call the specialized team for PICC insertion. The hospital has a protocol for device selection based on international and national guidelines, such as Standard Of Practice^7^, and national surveillance guidelines considering factors like intravenous therapy lasting longer than 6 days or medications incompatible with peripheral access, among others.

VAST performs an initial assessment to determine whether the PICC catheter can be inserted or if there are any contraindications. VAST maintains all records in a database using a medical chart abstraction tool with structured fields, in which data related to catheter insertion are recorded prospectively, including information on initial assessment and pre-, intra-, and post-procedure. In use since the group was established in 2011, this tool was not designed specifically for this study. Data related to PICC removal are subsequently collected by a nursing technician through active review of the patient’s electronic medical record. Although no formal data audit process is in place, the information recorded on the database corresponds directly to entries in the patients’ electronic medical records.

Ultrasound was used for all PICC screening before insertion and during the insertions. During the screening, the VAST nurse checked for catheter-to-vessel ration avoiding occupation above 40%. All insertions follow the maximum barrier procedure: the basilic was preferred as first choice, followed by brachial vein, and lastly the cephalic vein.

Most tip confirmations used the catheter with ECG technology. If the catheter did not have the technology for using a Sherlock™ device or if the patient had a pacemaker or other cardiovascular condition that could interfere with the ECG, X-ray was conducted to verify the catheter tip. In some cases, the path of the PICC tip was verified using “Navigation mode”, thus confirming that the catheter was located in the superior vena cava; due to interference on the final ECG, however, identifying the exact tip location was not possible. For these patients, X-ray was also necessary. 

### Data collection 

For convenience, data recorded from February 2020 to February 2022 were collected by exporting them to an Excel spreadsheet using the Research Electronic Data Capture (REDCap^®^) software. During the study period, 2,038 PICCs were inserted in COVID-19 patients. Of these, 27 catheters failed. Additionally, one PICC placement was interrupted during the procedure due to the patient’s clinical instability, two PICCs were removed because the tip was in the subclavian vein, two other PICCs were inserted in pediatric patients, and 945 PICCs lacked an outcome observation. Considering the inclusion criteria, these PICCs were excluded from the study, totaling 1,061 COVID-19 patients with PICC (Figure 1).

**Figure 1:**
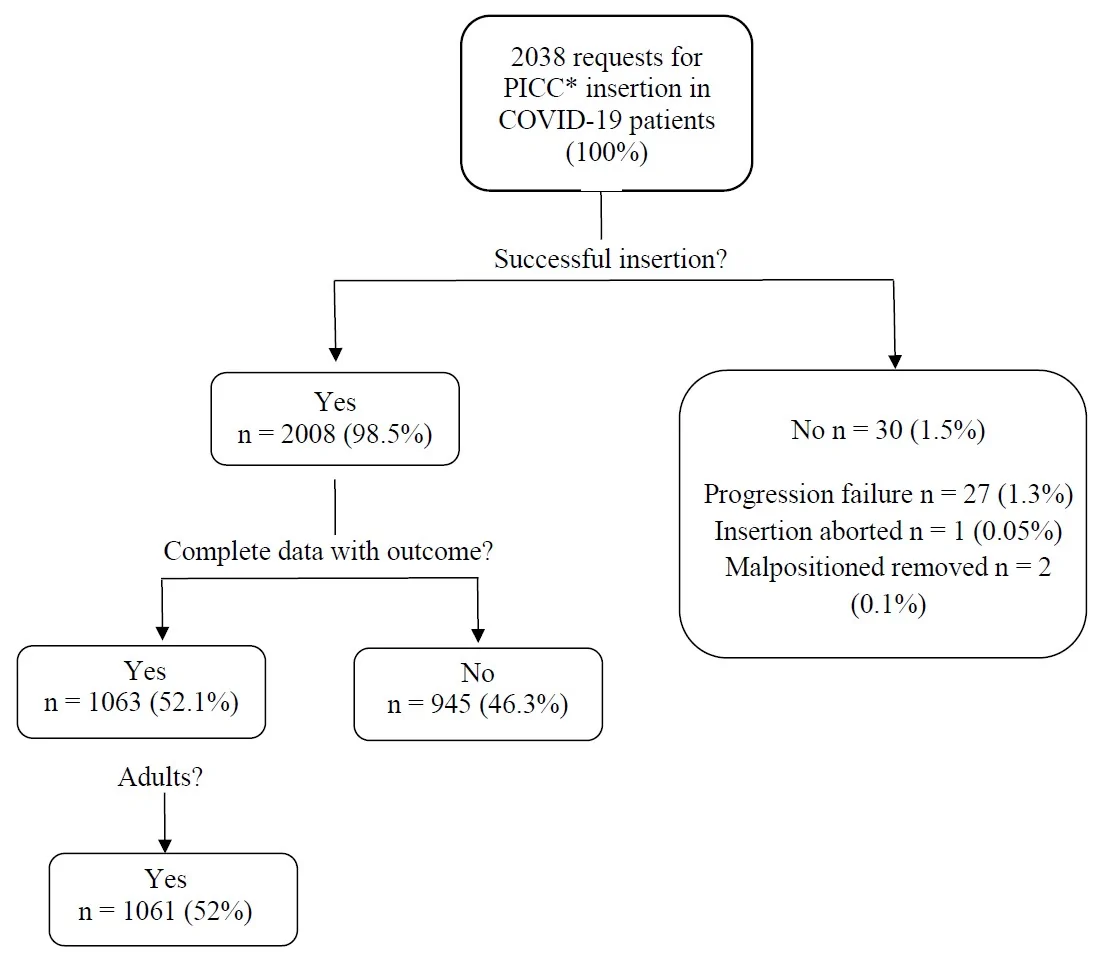
Flowchart of inclusion criteria

*PICC = Peripherally Inserted Central Catheter

### Study variables

The following variables were extracted using the REDCap^®^ software: Patient profile (critically ill or surgical patient); Deep Vein Thrombosis (DVT) prophylaxis and type of medication used; number of attempts until successful; vein chosen for puncture (basilic, axillary, cephalic, or brachial); percentage of catheter-to-vessel ratio calculated in ultrasound according to the size of selected vein and the type of catheter; Puncture zone, determined using the Zone Insertion Method (ZIM), ideally green, yellow, and red, as described in the literature^12^; Type of catheter: Power PICC^®^: single- (SL), double- (DL) and triple-lumen (TL) catheter. Valved double- and triple-lumen catheter; method for tip confirmation including Sherlock^TM^, X-ray, or fluoroscopy. When X-ray was used, the zone of the catheter tip in the SVC was also recorded (Zone A - lower third of the SVC, at the cavoatrial junction; Zone B and C lie in the middle and upper thirds of the SVC, respectively)^12^.

Cases were classified as progression failure when the catheter did not advance fully, as measured prior to the start of insertion, even after maneuvers like adjusting the patient’s head position were implemented. This failure is not related to insertion technique, as the vein was successfully cannulated and the guidewire threaded, and may instead be due to catheter obstruction caused by thrombosis or venous stenosis.

The following PICC outcomes were considered in this study: PICC removed at the end of intravenous therapy due to completion of IV therapy; or PICC is in good condition and maintained so that the patient can be discharged from the hospital with the catheter to complete the intravenous therapy at home, with home care. 

Others outcomes included if PICC was removed before intravenous therapy completion due to the following complications: accidental pull by the patient or nursing team during a procedure; thrombosis confirmed by an imaging report and Doppler ultrasound performed by the physician based on the presence of one of the following symptoms: axillary pain, limb edema, change in color, increase in arm circumference (more than 3 cm). 

Primary outcome concerned central line-associated bloodstream infection (CLABSI), defined by Centers for Disease Control and Prevention/National Healthcare Safety Network (CDC/NHSN) criteria as a laboratory-confirmed bloodstream infection occurring in a patient with a PICC in place or within 48 hours of device removal, with no other apparent source of infection^13^. CLABSI was chosen as the primary outcome due to its clinical relevance and impact on patient safety and catheter management.

Suspected bloodstream infection (SBSI) was included as a secondary outcome, defined as the presence of at least one of the following in patients with a central line: fever, ostium hyperemia, or elevated C-reactive protein (CRP), with no other apparent source of infection and a PICC in place for at least 48 hours. Although the institutional protocol does not require blood cultures or catheter tip analysis for all SBSI cases, most were submitted for blood culture analysis. 

The term negative outcomes was used as a composite endpoint, encompassing the occurrence of at least one of the following events: CLABSI or device-associated thrombosis resulting in unplanned catheter removal.

### Data analysis

Categorical data were expressed as absolute numbers and percentages, with the median and interquartile range of responses presented in tables. Quantitative variables were tested for normality using the Kolmogorov-Smirnov test. Non-parametric tests were adopted given the non-normal distribution of the sample. Relations between categorical variables or qualitative data were analyzed using Pearson’s chi-squared test or Fisher’s Exact Test as applicable. Mann-Whitney’s U test was used for comparison with ordinal or continuous variables. Logistic Regression was performed with variables associated with outcome. All statistical tests were performed on IBM SPSS Statistics, version 29 (Inc. Chicago, USA), at a significance level p < 0.05. 

### Ethical aspects

The present study was approved by the Human Research Ethics Committee of the study hospital (number 53585321.9.0000.5461) and conducted in compliance with the guidelines and regulatory standards of Resolution no. 466/2012, with provisions on human subject research. As data were retrospectively collected from medical records, no informed consent form was required. 

## Results

Patients presented a mean age of 68 years, and most were male. Most patients were hospitalized in ICUs when the PICC was requested. Diabetes Mellitus (DM) and systemic arterial hypertension were found in 7.1% and 9.1% of patients, respectively. The main reasons for PICC request were administration of antibiotics and irritating drugs and damage to the venous network. The catheter was pulled to adjust the position in 6.8% of patients who required a chest X-ray to confirm the PICC tip position. Table 1 presents the patient characteristics and factors associated with PICC failure.

**Table 1:** Characteristics of patients related to pre-procedure moments (n = 1061). São Paulo, SP, Brazil, 2025

Characteristic	Total N = 1061 (%)	Progression failure N = 27 (%)	p*	No confirmed complications n = 1030 (%)	Negative outcomes N = 31 (%)	p*
Male	747 (70.4%)	12 (44.4%)	0.004	722 (70.1%)	25 (80.6%)	0.205
Age	68 (56-79)	74 (60-87)	0.049	69 (57-81)	67 (61-75.5)	0.384
DM^†^ (no odds)	76 (7.2%)	0	0.251	71 (6.9%)	5 (16.1%)	0.049
SAH^‡^	97 (9.1%)	2 (7.4%)	1.000	92 (8.9%)	5 (16.1%)	0.171
Obesity	57 (5.4%)	1 (3.7%)	1.000	56 (5.4%)	1 (3.2%)	1.000
Solid tumor	19 (1.8%)	1 (3.7%)	0.398	19 (1.8%)	0	1.000
Subcutaneous heparin	855 (80.6%)	19 (70.4%)	< 0.001	832 (80.8%)	23 (74.2%)	0.426
Oral anticoagulant	42 (4%)	0	0.630	40 (3.9%)	2 (6.5%)	0.426
Pacemaker	9 (0.8%)	1 (3.7%)	0.223	9 (0.9%)	0	1.000
Previous DVT^§^	36 (3.4%)	3 (11.1%)	0.069	35 (3.4%)	1(3.2%)	1.000
Platelets	195,000 (145-255,000)	168,000 (137-237,000)	0.181	198,500 (148-257,000)	198,500 (143-306,000)	0.037
INR^||^	1.10 (1-1.2)	1.10 (0.9-1.2)	0.371	1.1 (1-1.2)	1.2 (1.1-1.2)	0.155
aPTT^¶^	1.02 (0.9-1.1)	1.05 (0.9-1.1)	0.813	1.02 (0.9-1.1)	1.0 (0.8-1.2)	0.648
Unit						
ED**	26(2.5%)	1 (3.7%)	0.679	26 (2.5%)	0	1.000
SEMI-Critical	135 (12.7%)	0	0.048	129 (12.5%)	6 (19.4%)	0.261
IU^††^	86 (8.1%)	3 (11.1%)	0.574	85 (8.3%)	1 (3.2%)	0.506
ICU^‡‡^	814 (76.7%)	23 (85.2%)	0.303	790 (76.7%)	24 (77.4%)	0.926

*p = p-value < 0.05 considered in the sample; ^†^DM = Diabetes Mellitus; ^‡^SAH = Systemic Arterial Hypertension; ^§^DVT = Deep Vein Thrombosis; ^||^INR = International Normalized Ratio; ^¶^aPTT = Activated Partial Thromboplastin Clotting Time; **ED = Emergency Department; ^††^IU = Inpatient Unit; ^‡‡^ICU = Intensive Care Unit. Statistical analyses used: Chi Square; Mann-Whitney U test

Main PICC outcomes included PICC removal after intravenous therapy completion in 663 (62.5%) patients, PICC removal due to death in 107 (10.1%) patients, PICC in good condition and maintained at hospital discharge to complete the intravenous therapy at home in 28 (2.6%) patients, and PICC removal due to the need for a PICC with more lumens in 13 (1.2%) patients. Additionally, 10 (0.9%) patients were transferred to another hospital and 12 due to other reasons (bleeding, malposition, among others). Adverse outcomes occurred in 228 (21.4%) patients who presented with some complication, including SBSI in 197 (18.5%) PICCs, which were removed before intravenous therapy completion. Among these patients with SBSI, blood culture tests were requested in 102 cases, 22 of which were positive for infection accounting for 2% of the total sample (CLABSI). Other complications observed included accidental pull in 17 (1.6%), occlusion in 5 (0.5%), and thrombosis in 9 (0.8%) cases. 

Some factors were associated with 31 negative outcomes (CLABSI and/or thrombosis), such as the use of X-ray (OR 3.264; 95%CI 1.555-6.851); however, X-ray confirmation was preferentially performed in more complex cases, such as patients with cardiovascular conditions and greater clinical severity. Thus, this association should be interpreted with caution. Catheter-to-vessel ratio ranging from 31% to 40% was associated with thrombosis p = 0.037 (OR 8.302; 95%CI: 1.664-41.417), Sherlock^TM^ tip confirmation was a protective factor for thrombosis p = 0.004 (OR 0.084; 95%CI: 0.10-0.672), and X-ray was associated with thrombosis p = 0.001 (OR 10.162; 95%CI: 2.517-41.022). Based on this analysis, risk factors for adverse outcomes were identified (Table 2).

**Table 2:** Analysis of variables predictable of negative outcome. São Paulo, SP, Brazil, 2025

Variables	B*	S.E.^†^	Wald	df^‡^	p^§^	Exp(B)^||^	95%CI^¶^ for EXP(B)^||^ Lower	95%CI^¶^ for EXP(B)^||^ Upper
DM**	0.960	0.510	3.535	1	0.060	2.611	0.960	7.101
Age	0.011	0.012	0.861	1	0.354	1.011	0.988	1.035
Dwell time	0.017	0.009	3.109	1	0.078	1.017	0.998	1.035
X-Ray	1.151	0.384	8.976	1	0.003	3.161	1.489	6.712

*B = Regression coefficient; ^†^S.E. = Standard error; ^‡^df = Degrees of Freedom; ^§^p = p-value < 0,05 considered in the sample; ^||^ExpB = Exponentiation of the coefficient; ^¶^CI = 95% Confidence interval for the Odds Ratio (OR); **DM = Diabetes Mellitus. Statistical analyses used: logistic regression

Given that 27 cases of unsuccessful insertion represent a substantial number, selected variables were analyzed to evaluate potential associations with progression failure. Being male was a protective factor against PICC progression failure (0.336 odds ratio (OR); 95% confidence interval (CI): 0.156-0.727), whereas requesting exchanging PICC for SBSI was associated with PICC progression failure (8.583 OR; 95%CI: 2.302-32.009). Being a clinical patient was also associated with PICC progression failure (13.550 OR; 95% CI: 4.267-43.035). Conversely, subcutaneous heparin administration was a protective factor against PICC progression failure (0.117 OR; 95%CI: 0.048-0.282). Lastly, first- (OR 6.565; 95%CI: 2.917-14.777), second- (OR 2.910; 95%CI: 1.070-7.882), and third-attempt (OR 9.538; 95%CI: 3.006-30.263) failures were also associated with PICC progression failure.

## Discussion

Although this study was conducted at a single institution with hospitalized COVID-19 patients, our findings on PICC-related risk factors, insertion practices, and vessel preservation strategies may have relevance for other high-acuity or isolated patient populations. However, the results should be interpreted cautiously as variations in institutional protocols, patient characteristics, and data completeness may limit their generalization.

Prevalence of comorbidities such as DM and systemic arterial hypertension in our study population was lower than 10%. Conversely, an Italian study on COVID-19 patients found that DM and systemic arterial hypertension were diagnosed in 20% and 53% of the patients included in the sample, respectively^14^. In a Korean study, systemic arterial hypertension and DM were diagnosed in over 40% of COVID-19 patients^3^. As the investigated hospital was private, the patients who are hospitalized or undergo procedures likely have favorable socioeconomic conditions. This difference might have influenced self-care and health monitoring. 

PICC use in critically ill patients was associated with a higher likelihood of occlusion^15^. In the present study, 100% of occlusion complications were worse in ICU patients.

Basilic vein presented the highest prevalence of puncture, followed by the brachial vein, as reported in a Korean study^3^. A Brazilian study also observed the highest prevalence of puncture in the basilic vein, albeit followed by the cephalic vein^16^. 

Valved PICC designs help limit blood backflow from the catheter tip into the lumen, thereby reducing the risk of catheter occlusion^17^. However, PICC removal due to occlusion occurred in only 0.5% of cases, considering that the most prevalent type of catheter was the open-ended catheter. A systematic review suggested that valved PICCs may be associated with fewer complications, although comparisons often involved heterogeneous PICC materials^17^. In a Brazilian study, the authors evaluated PICC outcomes in oncological patients, reporting infection in 13.6% and thrombosis in 1.94% of patients, with infection showing association with open-ended catheter use^16^.

The high prevalence (80%) of DL PICC in our study may be explained by the sample profile of critically ill patients (90%). DL and TL catheters may exacerbate infection complications^7^. A recent survey showed that most intensivists and hospitalists deem PICC insertion safer and more convenient for ICU patients than CVC^18^. This finding may support the choice of PICC for critically ill patients.

Median duration of catheter use was 13 days overall and 16 days in PICCs, with a negative outcome. In a previous study, the mean duration of catheter use in COVID-19 patients was approximately 14 days^3^. Longer catheter use (19 days vs. 15 days) has been significantly associated with adverse outcomes and infection^7^ ^,^ ^14^.

An analysis of US national data showed that central-line BSI (CLABSI) rates increased during the pandemic^19^. Moreover, catheter-related BSI (CRBSI) rates significantly increased from 1.89 to 5.53/1,000 admissions, particularly in ICU patients^20^. CLABSI rate was 2% in our study, in contrast to 1.8% in a French study^21^. In an Italian study on outcomes of PICC catheters in COVID-19 patients, the infection rate reached 5.8%^14^. Reported reasons for this increase included changes in line care due to pumps in hallways, prone positioning, staffing changes, reduced scrub-the-hub compliance, and less universal decolonization^22^. A systematic review found that most studies (17 of 21) reported higher CLABSI rates during the pandemic, with high workload, staff redeployment, and healthcare strain cited as factors potentially compromising infection prevention practices, including hand hygiene and line care bundles^23^.

A study showed that approximately 617 catheters were removed for culture tests, revealing catheter tip infection in 161 catheters^20^. In a French study, 14% (124) of the catheters were removed due to SBSI with infection confirmation in 8.1% (73) cases^7^. Fever and chills were good predictors of infection^7^. Here, only 11% (22 of 197) of SBSIs were ultimately confirmed, which could have led to some unnecessary removals and may have affected the accuracy of the reported CLABSI rates. Encouraging the collection of blood cultures whenever infection is suspected could help guide catheter removal decisions, highlighting the need to review and potentially update institutional protocols and policies for SBSI to avoid unnecessary catheter removals.

Over half of the catheters were removed after intravenous therapy completion, a positive outcome. This finding was similar to outcomes reported in other studies^7^ ^,^ ^19^ ^,^ ^24^. This positive outcome may have been due to the team of nurses experienced in catheter insertion and maintenance and management of complications who are exclusively dedicated to these functions^25^.

A French study showed PICC insertion failure in 128 procedures, accounting for 1% of total insertions^21^. Before the procedure, they evaluated the veins using the RaCeVA method^26^, which might have minimized failures, explaining why the failure rate (1%) was lower in their study than in ours (2.5%). 

We also explored factors related to catheter progression failure. In clinical patients, first- and third-attempts failure increased the odds of catheter progression failure. The number of needle punctures may be associated with difficult insertion, as shown in a Prague study which reported 44 patients with difficult insertions and complications during the 1-month follow-up^27^. Another protective factor against catheter progression failure may be anticoagulant use, as shown in a study with breast cancer patients undergoing chemotherapy, who exhibited fewer thrombosis events^28^. Our results corroborate the findings of the aforementioned studies. 

A Korean study reported catheter tip malpositioning in 12 cases (11 of which had tip position confirmed by X-ray), and 52% of these cases required catheter reinsertion^3^. Bedside electrocardiographic monitoring assists in accurate PICCs placement^6^. Real-time imaging during catheter placement may enable prompt intervention to prevent malposition; however, there is still little evidence of long-term outcomes of catheter use. In the present study, catheters requiring X-ray confirmation had an OR of 2.6 for negative outcomes.

Comorbidities and catheter-to-vessel ratio were not identified as risk factors for adverse outcomes. Catheter-to-vein ratio was associated with the risk of thrombosis in cancer patients, but not with infection or other non-malignant conditions^29^. A Brazilian study with 2,513 PICC patients reported an incidence of thrombosis in approximately 1.2% of patients^24^. Among the outcomes, thrombosis was confirmed in 0.8% of patients, as in a French study^21^.

We found that catheter tip confirmation by X-ray may be indirectly associated with thrombosis. An observational study reported that electrocardiography guidance was linked to a lower risk of catheter-related deep vein thrombosis and helped minimize radiation exposure^6^. However, this association should not be interpreted as causal. Radiographic confirmation is often reserved for patients in whom intracavitary electrocardiographic guidance is not feasible, who may present greater clinical complexity and higher baseline thrombotic risk. Thus, the observed association is likely indirect and may reflect confounding by indication rather than an independent effect of the confirmation method. A case series showed that catheter tips positioned using Sherlock were deeper than Zone B compared with X-ray confirmation^30^, which may explain the potential for optimal tip positioning and reduced complications. 

This study presents several limitations. First, its single-center design may limit the generalizability of the findings, as patient characteristics and the hospital’s financial resources to expand ICU capacity during the pandemic may not reflect other settings. Moreover, its retrospective nature limits analyses to available medical record data and may introduce bias in data collection and interpretation. Thus, the results should be interpreted with caution due to the exclusion of cases without recorded outcomes. Third, we were unable to analyze how many catheters the same patient had during hospitalization. A potential study limitation concerns the possible inaccuracy of CLABSI data, as the institutional protocol does not require blood cultures for all suspected bloodstream infections. Given the high number of catheter removals due to SBSI, the current protocol should be reviewed. As strengths, the study included a higher number of patients with COVID-19 and PICC, the VAST did not received new members during the pandemic, and every VAST member followed the same protocol as before the pandemic. 

## Conclusion

COVID-19 patients had outcomes akin to those reported in the literature, with catheter removal due therapy completion. PICC was removed due to SBSI in 18% of cases, with confirmed infection in 2%. Clinical profile and first-attempt failure were associated with catheter progression failure, increasing the odds of non-progression by 13.550 (95%CI 4.267-43.035) and 6.565 (95%CI 2.917-14.777), respectively. Using an X-ray to confirm tip position may be indirectly associated with negative outcomes. PICC is a safe choice for COVID-19 patients due to the bedside process allowing for equipment decontamination and real-time tip confirmation.

Nurses play a key role in improving PICC-related outcomes by identifying patient-specific risk factors, optimizing insertion practices, and promoting vessel preservation strategies. These practices may enhance patient safety and quality of care for hospitalized COVID-19 patients and may also be applicable to individuals requiring isolation or care during future pandemics. Further research is needed in the context of PICCs removed due to SBSI, particularly regarding implications for patient safety, healthcare costs, and vessel preservation.

## Data Availability

Datasets related to this article will be available upon request to the corresponding author.
