# Supplementary material for: Factors associated with failures and outcomes of Peripherally Inserted Central Catheters in hospitalized COVID-19 patients
Source: Rev Lat Am Enfermagem. 2026 Oct 23;34:e4970. doi: 10.1590/1518-8345.8298.4970 (PMC13706261; doi:10.1590/1518-8345.8298.4970)
Supplement: Translation

# Translation: Fatores associados a falhas e desfechos dos cateteres centrais de inserção periférica em pacientes hospitalizados com COVID-19

**DOI:** 10.1590/1518-8345.8298.4971

**Authors:** Amanda Saba, Jéssica Anastácia Silva Barbosa

**Affiliations:** 1 Universidade de São Paulo, São Paulo, SP, Brasil.; 2 Sociedade Beneficente de Senhoras Hospital Sírio-Libanês, São Paulo, SP, Brasil.

**Keywords:** Cateteres de Demora, Cuidados de Enfermagem, COVID-19, Infecções Relacionadas a Cateter, Falha de Equipamento, Pacientes Internados.

## Abstract

**Objetivo:**

analisar fatores associados à progressão do cateter e aos desfechos dos cateteres centrais de inserção periférica inseridos por enfermeiros em pacientes hospitalizados com COVID-19.

**Método:**

um estudo de coorte observacional foi realizado com pacientes hospitalizados com COVID-19, seguindo as diretrizes STROBE. Variáveis categóricas foram descritas como frequências absolutas e relativas; variáveis quantitativas foram expressas como mediana e intervalo interquartil. Testes não paramétricos foram aplicados devido à distribuição não normal. As associações foram analisadas usando os testes de qui-quadrado de Pearson ou exatos de Fisher. Foi realizada regressão logística para variáveis associadas aos desfechos.

**Resultados:**

um total de 1.061 cateteres centrais inseridos perifericamente foram analisados. As principais indicações eram de terapia antibiótica, e a maioria dos cateteres foi removida após a conclusão do tratamento. A falha na primeira tentativa e o perfil clínico do paciente estiveram associados à falha da progressão do cateter. A heparina subcutânea era um fator protetor. Entre cateteres centrais de inserção periférica inseridos com sucesso, a confirmação da ponta por raio-X do tórax e a razão cateter-vaso entre 31% e 40% estiveram associadas à trombose, enquanto a confirmação por Sherlock™ foi protetora.

**Conclusão:**

falha na progressão do cateter esteve associada a paciente clínico e falha na primeira tentativa de punção. Os resultados adversos estiveram associados à confirmação da ponta do raio-X e à razão cateter-vaso, enquanto o uso de Sherlock™ e a heparina subcutânea foram fatores protetores.

## Destaques

**(1)**A falha na primeira tentativa estava associada à falha de progressão.

**(2)**A heparina subcutânea está associada à redução da falha de progressão da PICC.

**(3)**O método usado para confirmação da ponta pode ser um fator protetor contra trombose.

**(4)**11% dos PICCs removidos para SBSI tinham CLABSI, destacando a necessidade de detectar BSI.

## Introdução

O cateter central de inserção periférica (PICC) apresenta uma alternativa ao cateter venoso central (CVC) e é amplamente utilizado para tratamentos de médio e longo prazo, tanto em internação quanto em ambulatorial^1^. Durante a pandemia da doença do coronavírus de 2019 (COVID-19), os pacientes - especialmente os da unidade de terapia intensiva (UTI) - frequentemente precisavam de acesso vascular confiável para terapia de suporte e coleta de sangue^2^. Essa situação ressaltou a necessidade de alocação eficiente de recursos e otimização das estratégias de saúde durante a pandemia^3^.

Um desafio crítico nesse período foi garantir o acesso vascular seguro e eficaz, minimizando o risco de contaminação aérea para os profissionais de saúde. A Declaração de Consenso GaVeCelt (*Gli Accessi Venosi Centrali a Lungo Termine*) discute as vantagens do uso de PICCs em relação aos CVCs em pacientes com dificuldades respiratórias graves, além daqueles que recebem pressão positiva contínua nas vias aéreas (CPAP) ou ventilação não invasiva (NVI) e aqueles que são propensos a anticoagulação ou anticoagulados. As PICC demonstraram reduzir o risco de complicações pleuropulmonares, evitar interferência no manejo respiratório e diminuir o risco de infecções no local de saída e exposição do operador a secreções traqueais, especialmente em pacientes traqueostomizados^4^.

Outro benefício importante dos PICCs é sua inserção sob orientação por ultrassom, que evita a necessidade de raios-X e reduz o risco de contaminação aérea. Isso é especialmente benéfico para a inserção ao lado do leito, eliminando a necessidade de transferir pacientes e, assim, reduzindo a exposição ao vírus para profissionais de saúde^2^. Além disso, o uso da colocação PICC guiada por Eletrocardiograma Intracavitário (IC-ECG) oferece confirmação da ponta em tempo real durante a inserção, tornando-a mais precisa, segura e econômica do que a orientação tradicional por raio-X^5^ ^-^ ^6^. A orientação do IC-ECG também elimina os atrasos associados à imagem por raios X, otimizando o uso do cateter^3^.

Apesar dessas vantagens, os PICCs não estão isentos de limitações. Por exemplo, a colocação de PICC guiada por ECG pode não ser adequada para pacientes com certas alterações no ECG, como fibrilação auricular, ou para aqueles com marcapassos^5^. Nesses casos, ainda pode ser necessário um raio-X para confirmar a colocação do cateter antes do uso. Além disso, fatores como tipo de terapia, duração e condições dos vasos devem ser cuidadosamente considerados ao selecionar o dispositivo mais adequado para cada paciente^5^. A remoção da PICC normalmente ocorre após a conclusão da terapia intravenosa, mas complicações como infecções na corrente sanguínea, trombose venosa ou oclusões podem exigir remoção prematura. Em um estudo francês, 38% dos cateteres foram removidos antes de concluir a terapia intravenosa^7^. Medidas preventivas durante a inserção, incluindo o uso de técnicas assépticas, proteção máxima da barreira e orientação por ultrassom, podem minimizar esses riscos. A manutenção contínua do cateter também requer formação contínua e treinamento dos profissionais de saúde, especialmente em relação à aderência, técnicas adequadas de lavagem e mudanças adequadas de curativos^8^.

Pacientes gravemente doentes com COVID-19, que são propensos a alterações na cascata de coagulação e hipercoagulação, apresentam alto risco de eventos trombóticos. Isso aumenta a complexidade do acesso vascular e torna as CVCs menos ideais devido ao risco de trombose pericateter e complicações adicionais como pneumotórax^9^ ^-^ ^10^.

Embora a fase aguda da pandemia tenha passado, as complicações de longo prazo da COVID-19 e o cuidado contínuo dos pacientes afetados pelo vírus destacam a necessidade de mais pesquisas sobre estratégias de acesso vascular. 

Apesar dos benefícios bem estabelecidos dos PICCs e da extensa literatura sobre complicações pós-inserção, menos atenção tem sido dada aos eventos ocorridos durante o próprio processo de inserção. Especificamente, quando a falha da progressão do cateter - quando o cateter não avança totalmente, mesmo após manobras - ocorre antes que o dispositivo atinja sua posição intravascular pretendida. Essa falha não só atrasa o tratamento, como também pode aumentar custos e pode exigir intervenções adicionais. Diante dos desafios contínuos e da necessidade de tomada de decisão baseada em evidências no manejo do cateter para pacientes com COVID-19, este estudo analisou os resultados dos cateteres centrais inseridos perifericamente (PICCs) por enfermeiros em pacientes hospitalizados com COVID-19 e fatores associados à falha da progressão do cateter.

## Método

### Design

Um estudo de coorte observacional foi realizado seguindo as diretrizes STROBE (*Strengthening the Reporting of Observational Studies in Epidemiology*)^11^. 

### Parâmetros do estudo

Este estudo foi realizado em um hospital de ensino e pesquisa de alta complexidade localizado na cidade de São Paulo, credenciado pela *Joint Commission International* (JCI) e com outras certificações. Este hospital responde a emergências com vários níveis de complexidade, oferecendo atendimento a pacientes com planos de saúde privados ou no contexto de parcerias público-privadas. O hospital conta com uma equipe especializada em acesso vascular (VAST) composta por enfermeiros registrados que inserem e mantêm PICCs, cateteres intravenosos periféricos guiados por ultrassom, avaliação semanal do PICC, entre outras atividades. A VAST tinha sete integrantes e contratou outros três profissionais devido à alta demanda durante a pandemia. Todos os integrantes possuíam certificado de qualificação para inserção e especialização no PICC, enquanto três possuíam mestrado.

### Participantes

Os participantes do estudo foram pacientes hospitalizados com COVID-19 com inserção de PICC, com 18 anos ou mais. Pacientes ambulatoriais e oncológicos, e PICC colocados por médicos vasculares foram excluídos deste estudo. Registros PICC com informações de desfecho incompletos foram excluídos da amostra final. Para pacientes com mais de uma colocação de PICC, todas as colocações foram consideradas.

### Solicitação e inserção do PICC

A equipe médica avalia a terapia intravenosa (duração e componentes) e as condições de acesso do paciente para chamar a equipe especializada para a inserção do PICC. O hospital possui um protocolo para seleção de dispositivos baseado em diretrizes internacionais e nacionais, como o Padrão de Prática^7^ e diretrizes nacionais de vigilância que consideram fatores como terapia intravenosa com duração superior a seis dias ou medicamentos incompatíveis com acesso periférico, entre outros.

O VAST realiza uma avaliação inicial para determinar se o cateter PICC pode ser inserido ou se há contraindicações. O VAST mantém todos os registros em um banco de dados usando uma ferramenta de abstração de prontuários médicos com campos estruturados, nos quais os dados relacionados à inserção de cateter são registrados prospectivamente, incluindo informações sobre avaliação inicial e pré, intra e pós-procedimento. Em uso desde que o grupo foi fundado em 2011, esta ferramenta não foi projetada especificamente para este estudo. Os dados relacionados à remoção do PICC são posteriormente coletados por um técnico de enfermagem por meio da revisão ativa do prontuário eletrônico do paciente. Embora não haja um processo formal de auditoria de dados, as informações registradas no banco de dados correspondem diretamente às entradas nos prontuários eletrônicos médicos dos pacientes.

O ultrassom foi utilizado em todas as triagens PICC antes da inserção e durante as inserções. Durante a triagem, a enfermeira do VAST verificou a ocupação do vaso, evitando ocupação acima de 40%. Todas as inserções seguem o procedimento de barreira máxima: a basílica foi preferida como primeira escolha, seguida pela veia braquial e, por fim, pela veia cefálica.

A maioria das confirmações de ponta utilizou o cateter com tecnologia de ECG. Se o cateter não tivesse tecnologia para usar um dispositivo Sherlock™ ou se o paciente tivesse um marcapasso ou outra condição cardiovascular que pudesse interferir no ECG, um raio-X era realizado para verificar a ponta do cateter. Em alguns casos, o caminho da ponta PICC foi verificado usando o “modo Navegação”, confirmando assim que o cateter estava localizado na veia cava superior; devido à interferência no ECG final, no entanto, não foi possível identificar a localização exata da ponta. Para esses pacientes, o raio-X também era necessário. 

### Coleta de dados 

Para maior conveniência, os dados registrados de fevereiro de 2020 a fevereiro de 2022 foram coletados exportando-os para uma planilha Excel usando o *software Research Electronic Data Capture* (REDCap^®^). Durante o estudo, 2.038 PICCs foram inseridos em pacientes com COVID-19. Destes, 27 cateteres falharam. Além disso, a colocação de um PICC foi interrompida durante o procedimento devido à instabilidade clínica do paciente, dois foram removidos porque a ponta estava na veia subclávia, outros dois foram inseridos em pacientes pediátricos, e 945 não apresentaram observação de desfecho. Considerando os critérios de inclusão, esses PICC foram excluídos do estudo, totalizando 1.061 pacientes com COVID-19 com PICC (Figura 1).

**Figura 1:** Fluxograma dos critérios de inclusão

*PICC = Cateter central de inserção periférica

### Variáveis de estudo

As seguintes variáveis foram extraídas usando o *software* REDCap^®^: perfil do paciente (doente crítico ou paciente cirúrgico); profilaxia e tipo de medicação utilizada para trombose venosa profunda (TVP); número de tentativas até sucesso; veia escolhida para punção (basílica, axilar, cefálica ou braquial); percentual da ocupação do vaso calculada em ultrassom de acordo com o tamanho da veia selecionada e o tipo de cateter; zona de punção, determinada usando o Método de Inserção de Zona (ZIM), idealmente verde, amarelo e vermelho, conforme descrito na literatura^12^; Tipo de cateter: *Power* PICC ^®^: cateter simples (SL), duplo (DL) e triplo lúmen (TL). cateter de duplo e triplo lúmen com válvulas; método para confirmação de ponta incluindo raio-X Sherlock^TM^ ou fluoroscopia. Quando o raio-X foi usado, a zona da ponta do cateter no SVC também foi registrada (Zona A - terço inferior do SVC, na junção cavoatrial; As Zonas B e C situam-se nos terços médio e superior do SVC, respectivamente)^12^.

Os casos foram classificados como falha de progressão quando o cateter não avançou totalmente, conforme medido antes do início da inserção, mesmo após manobras como ajustar a posição da cabeça do paciente foram implementadas. Essa falha não está relacionada à técnica de inserção, pois a veia foi canulada com sucesso e o fio-guia inserido, podendo ser devido à obstrução do cateter causada por trombose ou estenose venosa.

Os seguintes desfechos do PICC foram considerados neste estudo: PICC removido ao final da terapia intravenosa devido à conclusão da terapia intravenosa; ou PICC está em bom estado e mantido para que o paciente possa receber alta hospitalar com o cateter para completar a terapia intravenosa em casa, com cuidados domiciliares. 

Outros desfechos incluíram se o PICC foi removido antes da conclusão da terapia intravenosa devido às seguintes complicações: puxão acidental pelo paciente ou pela equipe de enfermagem durante o procedimento; trombose confirmada por um laudo de imagem e ultrassom Doppler realizado pelo médico com base na presença de um dos seguintes sintomas: dor axilar, edema do membro, mudança de cor, aumento da circunferência do braço (mais de 3 cm). 

O desfecho primário dizia respeito à infecção sanguínea associada à linha central (CLABSI), definida pelos critérios dos Centros de Controle e Prevenção de Doenças/Rede Nacional de Segurança em Saúde (CDC/NHSN), como uma infecção sanguínea confirmada em laboratório ocorrendo em um paciente com PICC no local ou dentro de 48 horas após a remoção do dispositivo, sem outra fonte aparente de infecção^13^. O CLABSI foi escolhido como desfecho principal devido à sua relevância clínica e impacto na segurança do paciente e no manejo do cateter.

A suspeita de infecção sanguínea (SBSI) foi incluída como desfecho secundário, definida como a presença de pelo menos um dos seguintes sintomas em pacientes com linha central: febre, hiperemia de óstio ou proteína C reativa elevada (PCR), sem outra fonte aparente de infecção e PICC no local por pelo menos 48 horas. Embora o protocolo institucional não exija hemoculturas ou análise da ponta do cateter para todos os casos de SBSI, a maioria foi submetida para análise de hemocultura. 

O termo “desfechos negativos” foi usado como um desfecho composto, abrangendo a ocorrência de pelo menos um dos seguintes eventos: CLABSI ou trombose associada ao dispositivo resultando em remoção não planejada do cateter.

### Análise de dados

Os dados categóricos foram expressos como números absolutos e percentagens, com a faixa mediana e interquartil de respostas apresentada em tabelas. Variáveis quantitativas foram testadas quanto à normalidade usando o teste de Kolmogorov-Smirnov. Testes não paramétricos foram adotados devido à distribuição não normal da amostra. As relações entre variáveis categóricas ou dados qualitativos foram analisadas usando o teste qui-quadrado de Pearson ou o Teste Exato de Fisher, conforme aplicável. O teste U de Mann-Whitney foi usado para comparação com variáveis ordinais ou contínuas. A regressão logística foi realizada com variáveis associadas ao resultado. Todos os testes estatísticos foram realizados no IBM SPSS Statistics, versão 29 (Inc. Chicago, EUA), em um nível de significância p < 0,05. 

### Aspectos éticos

Este estudo foi aprovado pelo Comitê de Ética em Pesquisa Humana do hospital de estudo (número 53585321.9.0000.5461) e feito em conformidade com as diretrizes e normas regulatórias da Resolução nº 466/2012, com disposições sobre pesquisa com sujeitos humanos. Como os dados eram coletados retroativamente a partir de prontuários médicos, não era necessário um formulário de consentimento informado. 

## Resultados

Os pacientes apresentaram uma idade média de 68 anos, sendo a maioria do sexo masculino. A maioria dos pacientes foi hospitalizada em UTI quando o PICC foi solicitado. Diabetes mellitus (DM) e hipertensão arterial sistêmica foram encontrados em 7,1% e 9,1% dos pacientes, respectivamente. Os principais motivos para o pedido de PICC foram a administração de antibióticos e medicamentos irritantes e danos à rede venosa. O cateter foi retirado para ajustar a posição em 6,8% dos pacientes que precisaram de um raio-X de tórax para confirmar a posição da ponta do PICC. A Tabela 1 apresenta as características dos pacientes e os fatores associados à falha da PICC.

**Tabela 1:** Características dos pacientes relacionadas aos momentos pré-procedimento (n = 1.061). São Paulo, SP, Brasil, 2025

Característica	Total N = 1061 (%)	Falha de progressão N = 27 (%)	p*	Sem complicações confirmadas n = 1030 (%)	Resultados negativos N = 31 (%)	p*
Masculino	747 (70,4%)	12 (44,4%)	0,004	722 (70,1%)	25 (80,6%)	0,205
Idade	68 (56-79)	74 (60-87)	0,049	69 (57-81)	67 (61-75,5)	0,384
DM^†^ (sem probabilidades)	76 (7,2%)	0	0,251	71 (6,9%)	5 (16,1%)	0,049
SAH^‡^	97 (9,1%)	2 (7,4%)	1,000	92 (8,9%)	5 (16,1%)	0,171
Obesidade	57 (5,4%)	1 (3,7%)	1,000	56 (5,4%)	1 (3,2%)	1,000
Tumor sólido	19 (1,8%)	1 (3,7%)	0,398	19 (1,8%)	0	1,000
Heparina subcutânea	855 (80,6%)	19 (70,4%)	< 0,001	832 (80,8%)	23 (74,2%)	0,426
Anticoagulante oral	42 (4%)	0	0,630	40 (3,9%)	2 (6,5%)	0,426
Marcapasso	9 (0,8%)	1 (3,7%)	0,223	9 (0,9%)	0	1,000
TVP anterior^§^	36 (3,4%)	3 (11,1%)	0,069	35 (3,4%)	1 (3,2%)	1,000
Plaquetas	195.000 (145-255.000)	168.000 (137-237.000)	0,181	198.500 (148-257.000)	198.500 (143-306.000)	0,037
INR^||^	1,10 (1-1,2)	1,10 (0,9-1,2)	0,371	1,1 (1-1,2)	1,2 (1,1-1,2)	0,155
aPTT^¶^	1,02 (0,9-1,1)	1,05 (0,9-1,1)	0,813	1,02 (0,9-1,1)	1,0 (0,8-1,2)	0,648
Unidade						
ED**	26 (2,5%)	1 (3,7%)	0,679	26 (2,5%)	0	1,000
SEMI-Crítico	135 (12,7%)	0	0,048	129 (12,5%)	6 (19,4%)	0,261
IU^††^	86 (8,1%)	3 (11,1%)	0,574	85 (8,3%)	1 (3,2%)	0,506
ICU^‡‡^	814 (76,7%)	23 (85,2%)	0,303	790 (76,7%)	24 (77,4%)	0,926

*p = Valor-p < 0.05 considerado na amostra; ^†^DM = Diabetes Mellitus; ^‡^SAH = Hipertensão Arterial Sistêmica; ^§^TVP = Trombose venosa profunda; ^||^INR = Razão Internacional Normalizada; ^¶^aPTT = Tempo de Coágulo Parcial Ativado; **ED = Pronto-Socorro; ^††^IU = Unidade de Internação; ^‡‡^UTI = Unidade de Terapia Intensiva. Análises estatísticas utilizadas: qui-quadrado; Teste Mann-Whitney U

Os principais desfechos incluíram remoção de PICC após a conclusão da terapia intravenosa em 663 (62,5%) pacientes, remoção de PICC devido à morte em 107 (10,1%) pacientes, PICC em bom estado e mantida na alta hospitalar para completar a terapia intravenosa em casa em 28 (2,6%) pacientes, e remoção devido à necessidade de PICC com mais lúmens em 13 (1,2%) pacientes. Além disso, 10 (0,9%) pacientes foram transferidos para outro hospital e 12 por outros motivos (sangramento, má disposição, entre outros). Desfechos adversos ocorreram em 228 (21,4%) pacientes que apresentaram alguma complicação, incluindo SBSI em 197 (18,5%) PICCs, que foram removidos antes da conclusão da terapia intravenosa. Entre esses pacientes com SBSI, foram solicitados testes de hemocultura em 102 casos, dos quais 22 foram positivos para infecção, representando 2% da amostra total (CLABSI). Outras complicações observadas incluíram puxão acidental em 17 (1,6%), oclusão em cinco (0,5%) e trombose em nove (0,8%) casos. 

Alguns fatores foram associados a 31 desfechos negativos (CLABSI e/ou trombose), como o uso de raio-X (OR 3,264; IC 95%: 1,555-6,851); no entanto, a confirmação por raio-X foi preferencialmente realizada em casos mais complexos, como pacientes com condições cardiovasculares e maior gravidade clínica. Portanto, essa associação deve ser interpretada com cautela. A razão cateter-vaso, variando de 31% a 40%, esteve associada à trombose p = 0,037 (OR 8,302; IC 95%: 1,664-41,417). A confirmação de ponta por Sherlock^TM^ foi um fator protetor para trombose p = 0,004 (OR 0,084; IC 95%: 0,10-0,672), e o raio-X foi associado à trombose p = 0,001 (OR 10,162; IC 95%: 2,517-41,022). Com base nessa análise, foram identificados fatores de risco para desfechos adversos (Tabela 2).

**Tabela 2:** Análise de variáveis preditoras de desfechos negativos. São Paulo, SP, Brasil, 2025

Variáveis	B*	E.P.^†^	Wald	gl^‡^	p^§^	Exp(B)^||^	IC 95%^¶^ para EXP(B)^||^ Inferior	IC 95%^¶^ para EXP(B)^||^ Superior
DM**	0,960	0,510	3,535	1	0,060	2,611	0,960	7,101
Idade	0,011	0,012	0,861	1	0,354	1,011	0,988	1,035
Tempo de permanência	0,017	0,009	3,109	1	0,078	1,017	0,998	1,035
Raio-X	1,151	0,384	8,976	1	0,003	3,161	1,489	6,712

*B = Coeficiente de regressão; ^†^E.P. = Erro-padrão; ^‡^gl = Graus de Liberdade; ^§^p = Valor-p < 0,05 considerado na amostra; ^||^ExpB = Exponenciação do coeficiente; ^¶^IC = Intervalo de confiança de 95% para a Razão de Chances (OR); **DM = Diabetes Mellitus. Análises estatísticas utilizadas: regressão logística

Considerando que 27 casos de inserção malsucedida representam um número substancial, variáveis selecionadas foram analisadas para avaliar possíveis associações com a falha da progressão. Ser homem foi um fator protetor contra a falha da progressão da PICC (OR 0,336; IC 95%: 0,156-0,727), enquanto o pedido de troca de PICC por SBSI estava associado à falha de progressão do PICC (8,583 OR; IC 95%: 2,302-32,009). Ser paciente clínico também esteve associado à falha de progressão do PICC (13,550 OR; IC 95%: 4,267-43,035). Por outro lado, a administração subcutânea de heparina foi um fator protetor contra a falha da progressão da PICC (0,117 OR; IC 95%: 0,048-0,282). Por fim, falhas na primeira (OR 6,565; IC 95%: 2,917-14,777), segunda (OR 2,910; IC 95%: 1,070-7,882), e na terceira tentativa (OR 9,538; IC 95%: 3,006-30,263) também estavam associadas à falha de progressão do PICC.

## Discussão

Embora este estudo tenha sido conduzido em uma única instituição com pacientes hospitalizados com COVID-19, nossos achados sobre fatores de risco relacionados ao PICC, práticas de inserção e estratégias de preservação de vasos podem ser relevantes para outras populações de pacientes de alta gravidade ou isoladas. No entanto, os resultados devem ser interpretados com cautela, pois variações nos protocolos institucionais, características dos pacientes e completude dos dados podem limitar sua generalização.

A prevalência de comorbidades como DM e hipertensão arterial sistêmica em nossa população estudada foi inferior a 10%. Por outro lado, um estudo italiano com pacientes com COVID-19 constatou que DM e hipertensão arterial sistêmica foram diagnosticadas em 20% e 53% dos pacientes incluídos na amostra, respectivamente^14^. Em um estudo coreano, hipertensão arterial sistêmica e DM foram diagnosticadas em mais de 40% dos pacientes com COVID-19^3^. Como o hospital investigado era privado, os pacientes hospitalizados ou submetidos a procedimentos provavelmente apresentam condições socioeconômicas favoráveis. Essa diferença pode ter influenciado o autocuidado e o monitoramento da saúde. 

O uso de PICC em pacientes críticos esteve associado a maior probabilidade de oclusão^15^. Neste estudo, 100% das complicações de oclusão foram piores em pacientes de UTI.

A veia basílica apresentou a maior prevalência de perfuração, seguida pela veia braquial, conforme relatado em um estudo coreano^3^. Um estudo brasileiro também observou a maior prevalência de perfuração na veia basílica, embora seguida pela veia cefálica^16^. 

Os projetos de PICC com válvula ajudam a limitar o retorno sanguíneo da ponta do cateter para o lúmen, reduzindo assim o risco de oclusão do cateter^17^. No entanto, a remoção da PICC devido à oclusão ocorreu em apenas 0,5% dos casos, considerando que o tipo mais prevalente de cateter era o cateter aberto. Uma revisão sistemática sugeriu que os PICC com válvula podem estar associados a menos complicações, embora as comparações frequentemente envolvessem materiais PICC heterogêneos^17^. Em um estudo brasileiro, os autores avaliaram os desfechos do PICC em pacientes oncológicos, relatando infecção em 13,6% e trombose em 1,94% dos pacientes, com infecção apresentando associação com o uso de cateter aberto^16^.

A alta prevalência (80%) de DL PICC em nosso estudo pode ser explicada pelo perfil amostral de pacientes críticos (90%). Cateteres DL e TL podem agravar complicações da infecção^7^. Uma pesquisa recente mostrou que a maioria dos médicos intensivistas e hospitalistas consideram a inserção PICC mais segura e conveniente para pacientes de UTI do que a CVC^18^. Esse achado pode apoiar a escolha do PICC para pacientes gravemente doentes.

A duração mediana do uso do cateter foi de 13 dias no total e 16 dias nos PICCs, com resultado negativo. Em um estudo anterior, a duração média do uso do cateter em pacientes com COVID-19 foi de aproximadamente 14 dias^3^. Uso prolongado do cateter (19 dias vs. 15 dias) esteve significativamente associada a desfechos adversos e infecção^7^ ^,^ ^14^.

Uma análise de dados nacionais dos EUA mostrou que as taxas de BSI de via central (CLABSI) aumentaram durante a pandemia^19^. Além disso, as taxas de BSI relacionadas a cateter (CRBSI) aumentaram significativamente de 1,89 para 5,53/1.000 internações, especialmente em pacientes de UTI^20^. A taxa de CLABSI foi de 2% em nosso estudo, em contraste com 1,8% em um estudo francês^21^. Em um estudo italiano sobre os resultados dos cateteres PICC em pacientes com COVID-19, a taxa de infecção atingiu 5,8%^14^. As razões relatadas para esse aumento incluíram mudanças no cuidado das linhas devido a bombas nos corredores, posicionamento deitado, mudanças na equipe de pessoal, redução da conformidade com o sistema de limpeza e menos descolonização universal^22^. Uma revisão sistemática constatou que a maioria dos estudos (17 de 21) relatou taxas mais altas de CLABSI durante a pandemia, com alta carga de trabalho, realocação de pessoal e pressão na saúde citados como fatores potencialmente comprometendo práticas de prevenção de infecções, incluindo higiene das mãos e pacotes de cuidados de linha^23^.

Um estudo mostrou que aproximadamente 617 cateteres foram removidos para testes de cultura, revelando infecção na ponta do cateter em 161 cateteres^20^. Em um estudo francês, 14% (124) dos cateteres foram removidos devido ao SBSI com confirmação de infecção em 8,1% (73) dos casos^7^. Febre e calafrios eram bons previsores de infecção^7^. Aqui, apenas 11% (22 de 197) dos SBSIs foram finalmente confirmados, o que pode ter levado a algumas remoções desnecessárias e pode ter afetado a precisão das taxas de CLABSI reportadas. Incentivar a coleta de hemoculturas sempre que houver suspeita de infecção pode ajudar a orientar as decisões sobre a remoção do cateter, destacando a necessidade de revisar e potencialmente atualizar protocolos e políticas institucionais para o SBSI, a fim de evitar remoções desnecessárias de cateteres.

Mais da metade dos cateteres foi removida após a conclusão da terapia intravenosa, um resultado positivo. Esse achado foi semelhante aos desfechos relatados em outros estudos^7^ ^,^ ^19^ ^,^ ^24^. Esse resultado positivo pode ter se devido à equipe de enfermeiros experientes em inserção e manutenção de cateteres e manejo de complicações, dedicados exclusivamente a essas funções^25^.

Um estudo francês mostrou falha na inserção do PICC em 128 procedimentos, representando 1% do total de inserções^21^. Antes do procedimento, eles avaliaram as veias usando o método RaCeVA^26^, o que pode ter minimizado as falhas, explicando por que a taxa de falha (1%) foi menor no estudo deles do que no nosso (2,5%). 

Também exploramos fatores relacionados à falha da progressão do cateter. Em pacientes clínicos, a falha na primeira e terceira tentativa aumentaram as chances de falha da progressão do cateter. O número de perfurações com agulha pode estar associado a inserções difíceis, como mostrado em um estudo de Praga que relatou 44 pacientes com inserções difíceis e complicações durante o acompanhamento de um mês^27^. Outro fator protetor contra a falha da progressão do cateter pode ser o uso de anticoagulantes, como demonstrado em um estudo com pacientes com câncer de mama em quimioterapia, que apresentaram menos eventos de trombose^28^. Nossos resultados corroboram os resultados dos estudos mencionados anteriormente. 

Um estudo coreano relatou mal posicionamento da ponta do cateter em 12 casos (11 dos quais tiveram a posição da ponta confirmada por raio-X), e 52% desses casos exigiram reinserção do cateter^3^. O monitoramento eletrocardiográfico à beira do leito auxilia na colocação precisa dos PICC^6^. Imagens em tempo real durante a colocação do cateter podem permitir intervenção rápida para prevenir má posição; No entanto, ainda há pouca evidência de resultados a longo prazo do uso do cateter. Neste estudo, cateteres que exigiram confirmação por raio-X tiveram um OR de 2,6 para desfechos negativos.

Comorbidades e ocupação de vaso não foram identificadas como fatores de risco para desfechos adversos. A razão cateter-veia estava associada ao risco de trombose em pacientes com câncer, mas não com infecção ou outras condições não malignas^29^. Um estudo brasileiro com 2.513 pacientes com PICC relatou incidência de trombose em aproximadamente 1,2% dos pacientes^24^. Entre os desfechos, a trombose foi confirmada em 0,8% dos pacientes, como em um estudo francês^21^.

Descobrimos que a confirmação da ponta do cateter por raio-X pode estar indiretamente associada à trombose. Um estudo observacional relatou que a orientação eletrocardiográfica estava ligada a um menor risco de trombose venosa profunda relacionada ao cateter e ajudava a minimizar a exposição à radiação^6^. No entanto, essa associação não deve ser interpretada como causal. A confirmação radiográfica é frequentemente reservada para pacientes em que a orientação eletrocardiográfica intracavitária não é viável, que podem apresentar maior complexidade clínica e maior risco trombótico basal. Assim, a associação observada provavelmente é indireta e pode refletir confusão por indicação, em vez de um efeito independentemente do método de confirmação. Uma série de casos mostrou que as pontas dos cateteres posicionadas com Sherlock™ eram mais profundas que a Zona B em comparação com a confirmação por raio-X^30^, o que pode explicar o potencial para posicionamento ideal da ponta e redução de complicações. 

Este estudo possui várias limitações. Primeiro, seu design de centro único pode limitar a generalização dos achados, já que as características dos pacientes e os recursos financeiros do hospital para expandir a capacidade da UTI durante a pandemia podem não refletir outros contextos. Além disso, sua natureza retrospectiva limita as análises aos dados disponíveis de prontuários médicos e pode introduzir viés na coleta e interpretação dos dados. Portanto, os resultados devem ser interpretados com cautela devido à exclusão de casos sem resultados registrados. Terceiro, não conseguimos analisar quantos cateteres o mesmo paciente recebeu durante a hospitalização. Uma possível limitação do estudo diz respeito à possível imprecisão dos dados CLABSI, já que o protocolo institucional não exige hemoculturas para todas as infecções suspeitas de corrente sanguínea. Dado o alto número de remoções de cateter devido à SBSI, o protocolo atual deve ser revisado. Como pontos fortes, o estudo incluiu um número maior de pacientes com COVID-19 e PICC, o VAST não recebeu novos membros durante a pandemia, e todos os membros seguiram o mesmo protocolo de antes da pandemia. 

## Conclusão

Pacientes com COVID-19 apresentaram desfechos semelhantes aos relatados na literatura, com remoção do cateter devido à conclusão da terapia. O PICC foi removido devido ao SBSI em 18% dos casos, apenas 2% do total de PICCs teve infecção confirmada. O perfil clínico e a falha na primeira tentativa de punção estiveram associados à falha da progressão do cateter, aumentando as chances de não progressão em 13,550 (IC 95% 4,267-43,035) e 6,565 (IC 95% 2,917-14,777), respectivamente. O uso de um raio-X para confirmar a posição da ponta pode estar indiretamente associado a resultados negativos. O PICC é uma escolha segura para pacientes com COVID-19 devido ao processo à beira do leito que permite a descontaminação de equipamentos e a confirmação da ponta em tempo real.

Os enfermeiros desempenham um papel fundamental na melhoria dos resultados relacionados ao PICC, identificando fatores de risco específicos de cada paciente, otimizando práticas de inserção e promovendo estratégias de preservação de vasos. Essas práticas podem melhorar a segurança do paciente e a qualidade do cuidado para pacientes hospitalizados com COVID-19 e podem ser aplicáveis a indivíduos que necessitem de isolamento ou cuidados durante futuras pandemias. Mais pesquisas são necessárias no contexto da remoção dos PICCs devido ao SBSI, especialmente em relação às implicações para a segurança do paciente, custos de saúde e preservação de vasos.

## Data Availability

Os conjuntos de dados relacionados a este artigo estarão disponíveis mediante solicitação ao autor correspondente.

# Translation: Factores asociados a fallas y desenlaces de los catéteres centrales de inserción periférica en pacientes hospitalizados con COVID-19

**DOI:** 10.1590/1518-8345.8298.4969

**Authors:** Amanda Saba, Jéssica Anastácia Silva Barbosa

**Affiliations:** 1 Universidade de São Paulo, São Paulo, SP, Brasil.; 2 Sociedade Beneficente de Senhoras Hospital Sírio-Libanês, São Paulo, SP, Brasil.

**Keywords:** Cateteres Permanentes, Cuidados de Enfermería, COVID-19, Infecciones Relacionadas con Catéteres, Falla de Equipo, Pacientes Internos.

## Abstract

**Objetivo:**

analizar los factores asociados con la progresión del catéter y los desenlaces de los catéteres centrales de inserción periférica colocados por enfermeros en pacientes hospitalizados con COVID-19.

**Método:**

se realizó un estudio de cohorte observacional con pacientes hospitalizados con COVID-19, siguiendo las directrices STROBE. Las variables categóricas se describieron mediante frecuencias absolutas y relativas; las variables cuantitativas se expresaron mediante la mediana y el rango intercuartil. Se aplicaron pruebas no paramétricas debido a la distribución no normal. Las asociaciones se analizaron utilizando la prueba de chi-cuadrado de Pearson o la prueba exacta de Fisher. Se realizó una regresión logística para las variables asociadas a los desenlaces.

**Resultados:**

se analizó un total de 1.061 catéteres centrales de inserción periférica. Las principales indicaciones fueron de terapia con antibióticos, y la mayoría de los catéteres se retiró una vez finalizado el tratamiento. La falla en el primer intento y el perfil clínico del paciente se asociaron al fracaso en la progresión del catéter. La heparina subcutánea fue un factor protector. Entre los catéteres centrales de inserción periférica insertados con éxito, la confirmación de la punta mediante radiografía de tórax y una relación catéter-vaso de entre el 31% y el 40% se asociaron con trombosis, mientras que la confirmación mediante Sherlock™ resultó protectora.

**Conclusión:**

la falla en la progresión del catéter se asoció con condiciones clínicas y con la falla en el primer intento. Los resultados adversos se asociaron a la confirmación radiográfica de la punta y la relación catéter-vaso, mientras que el uso de Sherlock™ y heparina subcutánea fueron factores protectores.

## Destacados

**(1)**La falla en el primer intento se asoció con la falta de progresión.

**(2)**La heparina subcutánea está asociada a la reducción de la falla de progresión del catéter PICC.

**(3)**El método utilizado para confirmar la punta puede ser un factor protector contra la trombosis.

**(4)**El 11% de los catéteres PICC retirados por SBSI tenían CLABSI, lo que subraya la necesidad de detectar BSI.

## Introducción

Los catéteres centrales de inserción periférica (PICC) ofrecen una alternativa al catéter venoso central (CVC) y se utilizan ampliamente para tratamientos a medio y largo plazo, tanto en la hospitalización como en servicios ambulatorios^1^. Durante la pandemia de la enfermedad por coronavirus (COVID-19) del 2019, los pacientes, especialmente aquellos en la unidad de cuidados intensivos (UCI), requirieron a menudo un acceso vascular confiable para la terapia de apoyo y la extracción de sangre^2^. Esta situación resaltó la necesidad de una asignación eficiente de recursos y la optimización de las estrategias de salud durante la pandemia^3^.

Un desafío crítico durante este período fue asegurar un acceso vascular seguro y eficaz, minimizando el riesgo de contaminación aérea para los profesionales sanitarios. La Declaración de Consenso de GaVeCelt (*Gli Accessi Venosi Centrali a Lungo Termine*) discute las ventajas del uso de PICC en comparación con los CVC en pacientes con dificultad respiratoria severa, así como en aquellos que reciben presión positiva continua en las vías respiratorias (CPAP) o ventilación no invasiva (VNI), y en aquellos propensos a la anticoagulación o anticoagulados. Se ha demostrado que los PICC reducen el riesgo de complicaciones pleuropulmonares, evitan la interferencia en el manejo respiratorio y disminuyen el riesgo de infecciones en el sitio de salida y la exposición del operador a las secreciones traqueales, especialmente en pacientes traqueostomizados^4^.

Otra ventaja importante de los PICC es su inserción guiada por ultrasonido, lo que elimina la necesidad de radiografías y reduce el riesgo de contaminación por vía aérea. Esto es especialmente beneficioso para la inserción a pie de cama, una vez que elimina la necesidad de trasladar a los pacientes y reduce así la exposición al virus para los profesionales sanitarios^2^. Además, la colocación de PICC guiada por Electrocardiograma Intracavitario (IC-ECG) ofrece confirmación de la punta en tiempo real durante la inserción, lo que la hace más precisa, segura y económica que la guía tradicional por rayos X^5^ ^-^ ^6^. La guía del IC-ECG también elimina los retrasos asociados con las imágenes de rayos X, lo que optimiza el uso del catéter^3^.

A pesar de estas ventajas, los PICC no están exentos de limitaciones. Por ejemplo, la colocación de PICC guiada por ECG puede no ser adecuada para pacientes con ciertas alteraciones en el ECG, como fibrilación auricular, o para aquellos con marcapasos^5^. En estos casos, aun puede ser necesaria una radiografía para confirmar la colocación del catéter antes de su uso. Además, factores como el tipo de terapia, la duración y el estado de los vasos deben considerarse con cuidado al seleccionar el dispositivo más adecuado para cada paciente^5^. La retirada del PICC suele producirse una vez finalizada la terapia intravenosa, pero complicaciones como infecciones del torrente sanguíneo, trombosis venosa u oclusiones pueden requerir su retirada prematura. En un estudio francés, se retiró el 38% de los catéteres antes de finalizar la terapia intravenosa^7^. Las medidas preventivas durante la inserción, incluido el uso de técnicas asépticas, la máxima protección de barrera y la guía ecográfica, pueden minimizar estos riesgos. El mantenimiento continuo del catéter también requiere formación continua y capacitación de los profesionales de la salud, especialmente en lo que respecta a la adhesión, las técnicas de lavado y los cambios de apósitos apropiados^8^.

Los pacientes con COVID-19 en estado grave, que son propensos a sufrir alteraciones en la cascada de coagulación e hipercoagulación, presentan un alto riesgo de eventos trombóticos. Esto aumenta la complejidad del acceso vascular y hace que los CVC sean menos ideales debido al riesgo de trombosis pericatéter y complicaciones adicionales como neumotórax^9^ ^-^ ^10^.

Si bien la fase aguda de la pandemia ha pasado, las complicaciones a largo plazo de la COVID-19 y el cuidado continuo de los pacientes afectados por el virus destacan la necesidad de más investigaciones sobre las estrategias de acceso vascular. 

A pesar de los beneficios bien establecidos de los PICC y de la extensa bibliografía sobre las complicaciones posteriores a la inserción, se ha prestado menos atención a los eventos que ocurren durante el propio proceso de inserción. En concreto, cuando la falla en la progresión del catéter -es decir, cuando el catéter no avanza completamente, incluso después de realizar maniobras-, se produce antes de que el dispositivo alcance su posición intravascular prevista. Esta falla no solo retrasa el tratamiento, sino que también puede aumentar los costos y puede requerir intervenciones adicionales. Dados los desafíos continuos y la necesidad de una toma de decisiones basada en evidencias en el manejo de catéteres para pacientes con COVID-19, este estudio analizó los resultados de los catéteres centrales de inserción periférica (PICC) colocados por enfermeros en pacientes hospitalizados con COVID-19 y los factores asociados a la falla en la progresión del catéter.

## Método

### Diseño

Se realizó un estudio de cohorte observacional siguiendo las directrices STROBE (*Strengthening the Reporting of Observational Studies in Epidemiology*)^11^. 

### Parámetros del estudio

Este estudio se llevó a cabo en un hospital de enseñanza e investigación de alta complejidad ubicado en la ciudad de São Paulo, acreditado por *Joint Commission International* (JCI) y con otras certificaciones. Este hospital responde a emergencias de diversa complejidad, brindando atención a pacientes con planes de seguro médico privados o en el contexto de asociaciones público-privadas. El hospital cuenta con un equipo especializado en acceso vascular (VAST) compuesto por enfermeros registrados que insertan y mantienen los PICC, catéteres intravenosos periféricos guiados por ultrasonido, realizan evaluaciones semanales de los PICC, entre otras actividades. El VAST contaba con siete miembros y contrató a tres profesionales más debido a la alta demanda durante la pandemia. Todos los miembros contaban con certificado de cualificación para la inserción y especialización en PICC, mientras que tres de ellos tenían máster.

### Participantes

Participaron en el estudio pacientes hospitalizados con COVID-19 con PICC, con 18 años o más. Se excluyeron los pacientes ambulatorios y oncológicos, y los PICC colocados por médicos vasculares. Se excluyeron de la muestra final los registros de PICC cuya información sobre el desenlace se encontraba incompleta. En el caso de pacientes con más de una colocación de PICC, se consideraron todas las colocaciones.

### Solicitud e inserción de PICC

El equipo médico evalúa la terapia intravenosa (duración y componentes) y las condiciones de acceso del paciente para avisar al equipo de especialistas para la inserción del PICC. El hospital cuenta con un protocolo para seleccionar los dispositivos basado en directrices internacionales y nacionales, como el Guía de Práctica^7^ y directrices nacionales de vigilancia que consideran factores como terapia intravenosa con duración superior a seis días o medicamentos incompatibles con acceso periférico, entre otras cosas.

El VAST realiza una evaluación inicial para determinar si se puede insertar un catéter PICC o si existen contraindicaciones. El VAST mantiene todos los registros en una base de datos utilizando una herramienta de extracción de registros médicos con campos estructurados, en los que se registran de forma prospectiva los datos relacionados con la inserción del catéter, incluida la información sobre la evaluación inicial y la atención previa, durante y posterior al procedimiento. En uso desde que se fundó el grupo en el 2011, esta herramienta no se diseñó específicamente para este estudio. Posteriormente, un técnico de enfermería recopila los datos relacionados con la extracción del PICC mediante la revisión activa del historial clínico electrónico del paciente. Aunque no haya un proceso formal de auditoría de datos, la información registrada en la base de datos corresponde directamente a las entradas en las historias clínicas electrónicas médicas de los pacientes.

En todas las pruebas de evaluación para PICC se utilizó ecografía antes y durante la inserción. Durante la evaluación, la enfermera del equipo VAST comprobó la ocupación del vaso, asegurándose de que fuera superior al 40%. Todas las inserciones se realizaron siguiendo el procedimiento de máxima barrera: se prefirió la vena basílica como primera opción, seguida de la vena braquial y, finalmente, de la vena cefálica.

La mayoría de las confirmaciones de punta utilizó el catéter con tecnología de ECG. Si el catéter carecía de la tecnología necesaria para utilizar un dispositivo Sherlock™, o si el paciente tenía un marcapasos u otra afección cardiovascular que pudiera interferir en el ECG, se realizaba una radiografía para comprobar la punta del catéter. En algunos casos, se verificó la trayectoria de la punta del PICC mediante el “modo de navegación”, confirmando así que el catéter estaba ubicado en la vena cava superior; sin embargo, debido a interferencias en el ECG final, no fue posible identificar la ubicación exacta de la punta. En el caso de estos pacientes, también fue necesaria una radiografía. 

### Recopilación de datos

Para una mayor conveniencia, los datos registrados de febrero del 2020 a febrero del 2022 se recopilaron y se exportaron a una hoja de cálculo de Excel utilizando el *software Research Electronic Data Capture* (REDCap^®^). Durante el estudio, se insertaron 2.038 PICC en pacientes con COVID-19. De estos, 27 catéteres fallaron. Además, se interrumpió la colocación de un PICC durante el procedimiento debido a la inestabilidad clínica del paciente, dos se retiraron porque la punta estaba en la vena subclavia, otros dos se insertaron en pacientes pediátricos, y en 945 casos no se observó ningún desenlace. Teniendo en cuenta los criterios de inclusión, estos PICC se excluyeron del estudio, lo que resultó en un total de 1.061 pacientes con COVID-19 con PICC (Figura 1)

**Figura 1:** Diagrama de flujo de los criterios de inclusión

*PICC = Catéter central de inserción periférica

### Variables de estudio

Se extrajeron las siguientes variables utilizando el *software* REDCap^®^: perfil del paciente (paciente crítico o quirúrgico); profilaxis y tipo de medicamento utilizado para la trombosis venosa profunda (TVP); número de intentos hasta lograr el éxito; vena elegida para la punción (basílica, axilar, cefálica o braquial); porcentaje de ocupación del vaso calculado por ultrasonido según el tamaño de la vena seleccionada y el tipo de catéter; zona de punción, determinada mediante el Método de Inserción de Zona (ZIM), idealmente verde, amarillo y rojo, como se describe en la literatura^12^; tipo de catéter: *Power* PICC ^®^: catéter monolumen (ML), doble (DL) y triple lumen (TL), catéter de doble y triple lumen con válvulas; método para confirmación de puntas que incluye radiografía Sherlock^TM^ o fluoroscopia. Cuando se utilizó radiografía, también se registró la zona de la punta del catéter en la VCS (Zona A: tercio inferior de la VCS, en la unión cavoauricular; las zonas B y C se ubican en los tercios medio y superior de la VCS, respectivamente)^12^.

Se calificaron como falla de progresión los casos en los que el catéter no avanzó completamente, según las mediciones realizadas antes del inicio de la inserción, incluso después de haber realizado maniobras como ajustar la posición de la cabeza del paciente. Esta falla no está relacionada con la técnica de inserción, ya que la vena se canuló correctamente y la guía se introdujo con éxito, por lo que puede deberse a una obstrucción del catéter causada por trombosis o estenosis venosa.

En este estudio se consideraron los siguientes desenlaces del PICC: Se retiró el PICC debido a la finalización de la terapia intravenosa; o bien, el catéter PICC se encuentra en buen estado y se mantiene para que el paciente pueda recibir el alta del hospital con el catéter para completar la terapia intravenosa en casa, con atención domiciliaria. 

Otros desenlaces incluyeron la retirada del PICC antes de completar la terapia intravenosa debido a las siguientes complicaciones: tracción accidental por parte del paciente o del personal de enfermería durante el procedimiento; trombosis confirmada por un informe de imágenes y ecografía Doppler realizada por el médico en función de la presencia de uno de los siguientes síntomas: dolor axilar, edema de la extremidad, cambio de color, aumento de la circunferencia del brazo (más de 3 cm). 

El desenlace primario se refería a la infección del torrente sanguíneo asociada a la vía central (CLABSI), definida por los criterios de los Centros para el Control y la Prevención de Enfermedades/Red Nacional de Seguridad en Salud (CDC/NHSN), como una infección del torrente sanguíneo confirmada por laboratorio que ocurre en un paciente con un PICC en el sitio o en las 48 horas posteriores a la extracción del dispositivo, sin otra fuente aparente de infección^13^. Se eligió el CLABSI como desenlace principal debido a su relevancia clínica y a su impacto en la seguridad del paciente y en el manejo del catéter.

La sospecha de infección del torrente sanguíneo (SBSI) se incluyó como desenlace secundario, definida como la presencia de al menos uno de los siguientes síntomas en pacientes con un catéter central: fiebre, hiperemia del ostium o proteína C reactiva elevada (PCR), sin otra fuente aparente de infección y PICC colocado durante al menos 48 horas. Si bien el protocolo institucional no exige hemocultivos ni análisis de la punta del catéter para todos los casos de SBSI, la mayoría se sometió a análisis de hemocultivo. 

El término “desenlaces negativos” se utilizó como un desenlace compuesto, que abarca la ocurrencia de al menos uno de los siguientes eventos: CLABSI o trombosis asociada a dispositivo que requiere la extracción no planificada del catéter.

### Análisis de los datos

Los datos categóricos se expresaron como números absolutos y porcentajes, y la mediana y el rango intercuartil de las respuestas se presentaron en tablas. Las variables cuantitativas se sometieron a pruebas de normalidad utilizando la prueba de Kolmogorov-Smirnov. Se adoptaron pruebas no paramétricas debido a la distribución no normal de la muestra. Las relaciones entre variables categóricas o datos cualitativos se analizaron mediante la prueba de chi-cuadrado de Pearson o la Prueba Exacta de Fisher, según correspondiera. Se utilizó la prueba U de Mann-Whitney para la comparación con variables ordinales o continuas. Se realizó una regresión logística utilizando variables asociadas al resultado. Todas las pruebas estadísticas se realizaron utilizando IBM SPSS Statistics, versión 29 (Inc. Chicago, EE. UU.), con un nivel de significancia de p < 0,05. 

### Aspectos éticos

Este estudio fue aprobado por el Comité de Ética de Investigación con Seres Humanos del hospital donde se realizó el estudio (número 53585321.9.0000.5461) y se llevó a cabo en conformidad con las directrices y normas reglamentarias de la Resolución n.º 466/2012, que trata sobre la investigación con sujetos humanos. Dado que los datos se recopilaron retrospectivamente de los historiales médicos, no fue necesario un formulario de consentimiento informado. 

## Resultados

Los pacientes tenían una edad media de 68 años, y la mayoría era hombre. La mayoría de los pacientes ingresó en la UCI cuando se solicitó la colocación de un PICC. Se detectó diabetes mellitus (DM) e hipertensión arterial sistémica en el 7,1% y el 9,1% de los pacientes, respectivamente. Los principales motivos para solicitar un PICC fueron la administración de antibióticos y medicamentos irritantes, y daños al sistema venoso. En el 6,8% de los pacientes, se retiró el catéter para ajustar su posición, y fue necesaria una radiografía de tórax para confirmar la posición de la punta del PICC. La Tabla 1 presenta las características de los pacientes y los factores asociados a la falla del PICC.

**Tabla 1:** Características de los pacientes relacionadas a los momentos previos al procedimiento (n = 1.061). São Paulo, SP, Brasil, 2025

Características	Total N = 1061 (%)	Falla de la progresión N = 27 (%)	p*	No se confirmaron complicaciones n = 1030 (%)	Resultados negativos N = 31 (%)	p*
Masculino	747 (70,4%)	12 (44,4%)	0,004	722 (70,1%)	25 (80,6%)	0,205
Edad	68 (56-79)	74 (60-87)	0,049	69 (57-81)	67 (61-75.5)	0,384
DM^†^ (sin probabilidades)	76 (7,2%)	0	0,251	71 (6,9%)	5 (16,1%)	0,049
SAH^‡^	97 (9,1%)	2 (7,4%)	1,000	92 (8,9%)	5 (16,1%)	0,171
Obesidad	57 (5,4%)	1 (3,7%)	1,000	56 (5,4%)	1 (3,2%)	1,000
Tumor sólido	19 (1,8%)	1 (3,7%)	0,398	19 (1,8%)	0	1,000
Heparina subcutánea	855 (80,6%)	19 (70,4%)	< 0,001	832 (80,8%)	23 (74,2%)	0,426
Anticoagulante oral	42 (4%)	0	0,630	40 (3,9%)	2 (6,5%)	0,426
Marcapasos	9 (0,8%)	1 (3,7%)	0,223	9 (0,9%)	0	1,000
TVP anterior^§^	36 (3,4%)	3 (11,1%)	0,069	35 (3,4%)	1 (3,2%)	1,000
Plaquetas	195.000 (145-255.000)	168.000 (137-237.000)	0,181	198.500 (148-257.000)	198.500 (143-306.000)	0,037
INR^||^	1,10 (1-1,2)	1,10 (0,9-1,2)	0,371	1,1 (1-1,2)	1,2 (1,1-1,2)	0,155
aPTT^¶^	1,02 (0,9-1,1)	1,05 (0,9-1,1)	0,813	1,02 (0,9-1,1)	1,0 (0,8-1,2)	0,648
Unidad						
ED**	26 (2,5%)	1 (3,7%)	0,679	26 (2,5%)	0	1,000
SEMICRÍTICO	135 (12,7%)	0	0,048	129 (12,5%)	6 (19,4%)	0,261
IU^††^	86 (8,1%)	3 (11,1%)	0,574	85 (8,3%)	1 (3,2%)	0,506
ICU^‡‡^	814 (76,7%)	23 (85,2%)	0,303	790 (76,7%)	24 (77,4%)	0,926

*p = Valor-p < 0,05 considerado en la muestra; ^†^DM = Diabetes Mellitus; ^‡^SAH = Hipertensión Arterial Sistémica; ^§^TVP = Trombosis venosa profunda; ^||^INR = Índice Internacional Normalizado; ^¶^aPTT = Tiempo de coagulación parcial activado; **ED = Sala de urgencias; ^††^IU = Unidad de ingreso; ^‡‡^UCI = Unidad de Cuidados Intensivos. Análisis estadísticos utilizados: chi-cuadrado; prueba U de Mann-Whitney

Los principales desenlaces incluyeron la retirada del PICC tras la finalización de la terapia intravenosa en 663 (62,5%) pacientes, la retirada del PICC debido a la muerte en 107 (10,1%) pacientes, PICC en buen estado y mantenido al momento del alta hospitalaria para completar la terapia intravenosa en el hogar en 28 (2,6%) pacientes, y la retirada debido a la necesidad de un PICC con más lúmenes en 13 (1,2%) pacientes. Además, 10 (0,9%) pacientes fueron trasladados a otro hospital y 12 por otros motivos (sangrado, mal posicionamiento, entre otros). Se produjeron desenlaces adversos en 228 (21,4%) pacientes que experimentaron alguna complicación, incluida SBSI en 197 (18,5%) PICC, que se retiraron antes de completar la terapia intravenosa. Entre estos pacientes con SBSI, se solicitaron pruebas de hemocultivo en 102 casos, de los cuales 22 fueron positivos para infección, lo que representa el 2% de la muestra total (CLABSI). Otras complicaciones observadas incluyeron tracción accidental en 17 casos (1,6%), oclusión en cinco casos (0,5%) y trombosis en nueve casos (0,8%). 

Algunos factores se asociaron a 31 desenlaces negativos (CLABSI o trombosis), como el uso de radiografía (OR 3,264; IC 95%: 1,555-6,851); sin embargo, la confirmación mediante radiografía se realizó preferentemente en casos más complejos, como pacientes con afecciones cardiovasculares y mayor gravedad clínica. Por lo tanto, esta asociación debe interpretarse con precaución. La relación catéter-vaso, que osciló entre el 31% y el 40%, se asoció a trombosis p = 0,037 (OR 8,302; IC 95%: 1,664-41,417). La confirmación de la punta con Sherlock^TM^ fue un factor protector contra la trombosis p = 0,004 (OR 0,084; IC 95%: 0,10-0,672), y la radiografía se asoció a trombosis p = 0,001 (OR 10,162; IC 95%: 2,517-41,022). Con base en este análisis, se identificaron factores de riesgo para desenlaces adversos (Tabla 2).

**Tabla 2:** Análisis de variables predictoras de desenlace negativo. São Paulo, SP, Brasil, 2025

Variables	B*	E.P.^†^	Wald	gl^‡^	p^§^	Exp(B)^||^	IC 95%^¶^ para EXP(B)^||^ Inferior	IC 95%^¶^ para EXP(B)^||^ Superior
DM**	0,960	0,510	3,535	1	0,060	2,611	0,960	7,101
Edad	0,011	0,012	0,861	1	0,354	1,011	0,988	1,035
Duración de la estancia	0,017	0,009	3,109	1	0,078	1,017	0,998	1,035
Radiografía	1,151	0,384	8,976	1	0,003	3,161	1,489	6,712

*B = Coeficiente de regresión; ^†^E.P. = Error estándar; ^‡^gl = Grados de Libertad; ^§^p = valor-p < 0,05 considerado en la muestra; ^||^ExpB = Exponenciación del coeficiente; ^¶^IC = Intervalo de confianza del 95% para la Razón de Probabilidades (OR); **DM = Diabetes Mellitus. Análisis estadísticos utilizados: regresión logística

Dado que 27 casos de inserción fallida representan una cantidad considerable, se analizaron variables seleccionadas para evaluar posibles asociaciones con la falla de la progresión. Ser hombre fue un factor protector contra el fracaso de la progresión del PICC (OR 0,336; IC 95%: 0,156-0,727), mientras que la solicitud de reemplazar el PICC debido a SBSI se asoció con el fracaso de la progresión del PICC (8,583 OR; IC del 95%: 2,302-32,009). Ser un paciente clínico también se asoció con el fracaso de la progresión del PICC (13,550 OR; IC 95%: 4,267-43,035). Por otra parte, la administración subcutánea de heparina fue un factor protector contra el fracaso de la progresión del PICC (0,117 OR; IC 95%: 0,048-0,282). Finalmente, las fallas en el primer (OR 6,565; IC 95%: 2,917-14,777), segundo (OR 2,910; IC 95%: 1,070-7,882), y en el tercer intento (OR 9,538; IC 95%: 3,006-30,263) también se asociaron con el fracaso de la progresión del PICC.

## Discusión

Aunque este estudio se realizó en una sola institución con pacientes hospitalizados por COVID-19, nuestros hallazgos sobre los factores de riesgo relacionados con los catéteres PICC, las prácticas de inserción y las estrategias de preservación vascular pueden ser relevantes para otras poblaciones de pacientes en estado crítico o aislados. Sin embargo, los resultados deben interpretarse con precaución, ya que las variaciones en los protocolos institucionales, las características de los pacientes y la exhaustividad de los datos pueden limitar su generalización.

La prevalencia de comorbilidades como la DM y la hipertensión arterial sistémica en nuestra población de estudio fue inferior al 10%. Por otra parte, en un estudio italiano con pacientes con COVID-19 se diagnosticó DM e hipertensión arterial sistémica en el 20% y en el 53% de los pacientes incluidos en la muestra, respectivamente^14^. En un estudio coreano, se diagnosticó hipertensión arterial sistémica y DM en más del 40% de los pacientes con COVID-19^3^. Dado que el hospital investigado era privado, es probable que los pacientes hospitalizados o sometidos a procedimientos tuvieran condiciones socioeconómicas favorables. Esta diferencia puede haber influido en el autocuidado y en el monitoreo de la salud. 

El uso de PICC en pacientes en estado crítico se asoció a una mayor probabilidad de oclusión^15^. En este estudio, el 100% de las complicaciones por oclusión fueron peores en los pacientes de la UCI.

La vena basílica mostró la mayor prevalencia de perforación, seguida de la vena braquial, como se informó en un estudio coreano^3^. Un estudio brasileño también observó la mayor prevalencia de perforación en la vena basílica, aunque seguida de la vena cefálica^16^. 

Los diseños de PICC con válvulas ayudan a limitar el retorno de sangre desde la punta del catéter al lumen, lo que reduce el riesgo de oclusión del catéter^17^. Sin embargo, la retirada del catéter PICC debido a oclusión se produjo en tan solo el 0,5% de los casos, teniendo en cuenta que el tipo de catéter más frecuente era el catéter abierto. Una revisión sistemática sugirió que los PICC con válvula pueden estar asociados con menos complicaciones, aunque las comparaciones a menudo implicaron materiales PICC heterogéneos^17^. En un estudio brasileño, los autores evaluaron los desenlaces del PICC en pacientes oncológicos y reportaron infección en el 13,6% y trombosis en el 1,94% de los pacientes, lo que revela una asociación de la infección con el uso de catéter abierto^16^.

La alta prevalencia (80%) de DL PICC en nuestro estudio se puede explicar por el perfil de la muestra de pacientes en estado crítico (90%). Los catéteres DL y TL pueden empeorar las complicaciones de la infección^7^. Una investigación reciente demostró que la mayoría de los intensivistas y médicos hospitalarios considera que la inserción de PICC es más segura y conveniente para los pacientes de la UCI que la de CVC^18^ ^).^. Este hallazgo podría respaldar la elección de un catéter PICC para pacientes en estado crítico.

La duración media del uso del catéter fue de 13 días en total y de 16 días para los PICC, con resultado negativo. En un estudio anterior, la duración promedio del uso de catéteres en pacientes con COVID-19 fue de aproximadamente 14 días^3^. El uso prolongado del catéter (19 días vs. 15 días) se asoció significativamente con resultados adversos e infección^7^ ^,^ ^14^.

Un análisis de datos nacionales de EE. UU. mostró que las tasas de BSI de vía central (CLABSI) aumentaron durante la pandemia^19^. Además, las tasas de BSI relacionadas con catéter (CRBSI) aumentaron significativamente de 1,89 a 5,53/1.000 hospitalizaciones, especialmente en pacientes de UCI^20^. La tasa de CLABSI fue del 2% en nuestro estudio, en contraste con el 1,8% en un estudio francés^21^. En un estudio italiano sobre los resultados de los catéteres PICC en pacientes con COVID-19, la tasa de infección alcanzó el 5,8%^14^. Las razones reportadas para este aumento incluyeron cambios en el cuidado de las líneas debido a las bombas en los pasillos, la posición de los pacientes acostados, cambios en el personal, menor cumplimiento con el sistema de limpieza y una descolonización menos universal^22^. Una revisión sistemática constató que la mayoría de los estudios (17 de 21) reportó tasas más altas de CLABSI durante la pandemia, con alta carga laboral, reasignación de personal y presión en el ámbito sanitario mencionados como factores que pueden afectar las prácticas de prevención de infecciones, incluida la higiene de las manos y paquetes de medidas de seguridad^23^.

Un estudio mostró que aproximadamente 617 catéteres fueron retirados para análisis de cultivo, y se constató infección de la punta en 161 catéteres^20^. En un estudio francés, el 14% (124) de los catéteres fueron retirados debido a SBSI con infección confirmada en el 8,1% (73) de los casos^7^. Fiebre y escalofríos eran buenos predictores de infección^7^. En este caso, solo el 11% (22 de 197) de los SBSI fueron finalmente confirmados, lo que puede haber dado lugar a algunas retiradas innecesarias y puede haber afectado la precisión de las tasas de CLABSI notificadas. Fomentar la toma de muestras para hemocultivos siempre que se sospeche una infección puede ayudar a orientar las decisiones sobre la retirada del catéter, lo que subraya la necesidad de revisar y, posiblemente, actualizar los protocolos y las políticas institucionales para el SBSI, con el fin de evitar retiradas innecesarias de catéteres.

Más de la mitad de los catéteres se retiró tras finalizar la terapia intravenosa, lo que supone un resultado positivo. Este hallazgo fue similar a los desenlaces reportados en otros estudios^7^ ^,^ ^19^ ^,^ ^24^. Este resultado positivo puede deberse al equipo de enfermeros con amplia experiencia en la inserción y mantenimiento de catéteres y manejo de complicaciones, dedicados exclusivamente a estas funciones^25^.

Un estudio francés mostró fallas en la inserción de PICC en 128 procedimientos, lo que representa el 1% del total de inserciones^21^. Antes del procedimiento, evaluaron las venas utilizando el método RaCeVA^26^, lo que puede haber minimizado las fallas, lo que explica por qué la tasa de fallas (1%) fue menor en su estudio que en el nuestro (2,5%). 

También exploramos factores relacionados con la falla en la progresión del catéter. En pacientes clínicos, el fracaso en el primer y tercer intento aumentó las probabilidades de que fallara la progresión del catéter. El número de perforaciones con aguja puede estar asociado a inserciones difíciles, como lo demuestra un estudio de Praga que reportó 44 pacientes con inserciones difíciles y complicaciones durante un mes de seguimiento^27^. Otro factor protector contra el fracaso en la progresión del catéter puede ser el uso de anticoagulantes, como se demostró en un estudio con pacientes con cáncer de mama sometidas a quimioterapia, que experimentaron menos eventos trombóticos^28^. Nuestros resultados corroboran los resultados de los estudios mencionados anteriormente. 

Un estudio coreano reportó un mal posicionamiento de la punta del catéter en 12 casos (11 de los cuales tenían la posición de la punta confirmada por radiografía), y el 52% de estos casos requirió la reinserción del catéter^3^. La monitorización electrocardiográfica a pie de cama ayuda a la colocación precisa de los PICC^6^. La obtención de imágenes en tiempo real durante la colocación del catéter permite una intervención rápida para prevenir una mala posición; sin embargo, aún hay poca evidencia sobre los resultados a largo plazo del uso del catéter. En este estudio, los catéteres que requirieron confirmación radiográfica tuvieron una OR de 2,6 para desenlaces negativos.

Las comorbilidades y la ocupación vascular no se identificaron como factores de riesgo de desenlaces adversos. La relación catéter-vena se asoció con el riesgo de trombosis en pacientes con cáncer, pero no con infección u otras afecciones no malignas^29^. Un estudio brasileño con 2.513 pacientes con PICC reportó una incidencia de trombosis en aproximadamente el 1,2% de los pacientes^24^. Entre los desenlaces, se confirmó la trombosis en el 0,8% de los pacientes, como en un estudio francés^21^.

Constatamos que la confirmación radiológica de la punta del catéter puede estar indirectamente asociada a la trombosis. Un estudio observacional informó que la guía electrocardiográfica se asoció con un menor riesgo de trombosis venosa profunda relacionada con el catéter y ayudó a minimizar la exposición a la radiación^6^. Sin embargo, esta asociación no debe interpretarse como causal. La confirmación radiográfica suele reservarse para pacientes en los que no es factible la guía electrocardiográfica intracavitaria, que pueden presentar una mayor complejidad clínica y un mayor riesgo trombótico basal. Por lo tanto, es probable que la asociación observada sea indirecta y que refleje un sesgo de indicación, en lugar de un efecto independiente del método de confirmación. Una serie de casos mostró que las puntas de los catéteres posicionadas con Sherlock™ eran más profundas que la Zona B en comparación con la confirmación radiográfica^30^, lo que puede explicar el potencial para posicionamiento ideal de la punta y la reducción de complicaciones. 

Este estudio tiene varias limitaciones. En primer lugar, su diseño de centro único puede limitar la generalización de los hallazgos, ya que las características de los pacientes y los recursos financieros del hospital para ampliar la capacidad de la UCI durante la pandemia pueden no reflejar otros contextos. Además, su carácter retrospectivo limita los análisis a los datos disponibles en los historiales médicos y puede introducir sesgos en la recopilación e interpretación de los datos. Por lo tanto, los resultados deben interpretarse con precaución debido a la exclusión de los casos sin resultados registrados. En tercer lugar, no pudimos analizar cuántos catéteres recibió el mismo paciente durante su hospitalización. Una posible limitación del estudio se refiere a la posible imprecisión de los datos de CLABSI, ya que el protocolo institucional no exige hemocultivos para todas las infecciones del torrente sanguíneo sospechosas. Dado el elevado número de retiradas de catéteres debido a SBSI, el protocolo actual debería revisarse. Como puntos fuertes, el estudio incluyó un mayor número de pacientes con COVID-19 y PICC, el hecho de que el equipo VAST no admitiera nuevos miembros durante la pandemia y que todos los miembros siguieran el mismo protocolo que antes de la pandemia. 

## Conclusión

Los pacientes con COVID-19 presentaron desenlaces similares a los descritos en la literatura, con retirada del catéter debido a la finalización de la terapia. En el 18% de los casos se retiró el catéter PICC debido a SBSI, y en el 2% se confirmó la infección. El perfil clínico y el fracaso en el primer intento se asociaron con el fracaso de la progresión del catéter, lo que aumenta las probabilidades de no progresión en 13.550 (IC 95 %: 4,267-43,035) y 6,565 (IC 95 %: 2,917-14,777), respectivamente. El uso de radiografía para confirmar la posición de la punta puede estar indirectamente asociado con resultados negativos. El PICC es una opción segura para los pacientes con COVID-19 debido al procedimiento que se realiza a pie de cama, el cual permite la descontaminación de los equipos y la confirmación de la punta del catéter en tiempo real.

Los enfermeros desempeñan un papel fundamental en la mejora de los resultados relacionados con los catéteres PICC, al identificar los factores de riesgo específicos de cada paciente, optimizar las prácticas de inserción y promover estrategias que preserven los vasos sanguíneos. Estas prácticas pueden mejorar la seguridad del paciente y la calidad del cuidado para los pacientes hospitalizados con COVID-19 y pueden ser aplicables a las personas que requieran aislamiento o cuidados durante futuras pandemias. Es necesario realizar más investigaciones en el contexto de la retirada de los PICC debido a SBSI, especialmente en lo que respecta a las implicaciones para la seguridad del paciente, los costos de salud y la preservación de los vasos sanguíneos.

## Data Availability

Los conjuntos de datos relacionados con este artículo estarán disponibles previa solicitud al autor correspondiente.
